# TAE-IDS: a trust-aware explainable intrusion detection framework using attention-based meta-ensemble learning with blockchain validation

**DOI:** 10.3389/frai.2026.1903112

**Published:** 2026-08-21

**Authors:** S. Antony Joseph Raj, M. Madiajagan

**Affiliations:** School of Computer Science and Engineering, Vellore Institute of Technology, Vellore, Tamil Nadu, India

**Keywords:** attention, blockchain, explainable AI, intrusion detection, meta-ensemble, SHAP, trust-aware security

## Abstract

Recent intrusion detection systems (IDS) increasingly rely on machine learning (ML) and deep learning techniques to detect sophisticated cyberattacks. However, many existing frameworks still suffer from limited explainability, black-box decision-making, and the absence of secure trust verification mechanisms for intrusion records. To address these challenges, this paper proposes TAE-IDS, a Trust-Aware Explainable Intrusion Detection Framework that integrates attention-based meta-ensemble learning, SHapley Additive exPlanations (SHAP)-driven explainability, and blockchain-inspired tamper-evident validation within a unified cybersecurity architecture. The proposed framework employs heterogeneous base classifiers, namely Logistic Regression (LR), Extra Trees (ET), and XGBoost (XGB), to capture diverse network traffic characteristics. Uncertainty-aware meta-features, including logits, confidence scores, and entropy representations, are extracted from the base learners and processed by an adaptive Bidirectional Long Short-Term Memory (BiLSTM) attention-based meta-classifier for contextual intrusion reasoning and adaptive ensemble aggregation. To enhance transparency and analyst trust, SHAP-based explainability is incorporated to provide both global and local interpretations of intrusion predictions. Furthermore, a blockchain-inspired tamper-evident validation mechanism based on SHA-256 cryptographic hashing is integrated to enable tamper-proof intrusion logging, immutable auditing, and secure forensic verification of IDS outputs. The proposed framework was evaluated on the UNSW-NB15 and CICIDS2017 benchmark datasets under both binary and multiclass intrusion detection settings. Experimental results demonstrate that TAE-IDS achieves strong intrusion detection performance, interpretable intrusion reasoning, and effective blockchain-assisted tamper-evident validation on the evaluated benchmark datasets. The integration of explainable artificial intelligence (XAI) and blockchain-assisted validation enhances transparency, forensic traceability, and the integrity of intrusion records while providing a foundation for future validation in operational network environments.

## 1 Introduction

The rapid growth of cloud computing environments, Internet of Things (IoT) devices, intelligent communication systems, and interconnected digital infrastructures has significantly increased the vulnerability of modern networks to sophisticated cyberattacks. Attacks like distributed denial-of-service (DDoS), ransomware, reconnaissance, infiltration, botnets, and advanced persistent threats (APTs) are common in modern network environments and jeopardize the availability, confidentiality, and integrity of vital information systems. As a result, intrusion detection systems (IDS), especially network intrusion detection systems (NIDS), are now vital parts of modern cybersecurity infrastructures for constantly keeping an eye on network activity and spotting unusual or hostile activity (Ferrag et al., 2020).

Since they typically rely on predefined attack signatures and physically created criteria, traditional signature-based intrusion detection techniques are becoming less and less effective against active and earlier undetected attacks. Because machine learning (ML) and deep learning-based intrusion detection frameworks can recognize complicated traffic patterns and distinguish between malicious and benign network behaviors, they have been generally adopted to address these constraints (Ferrag et al., 2020). Current ML-driven IDS architectures mostly employ single classifiers or ensemble learning methods. Single classifiers are computationally efficient, but they often have a limited ability to simplify in a variety of traffic scenarios. Ensemble learning approaches improve prediction robustness by integrating multiple learners to use their complementary strengths and reduce classification variance (Thakkar and Lohiya, 2023; Thockchom et al., 2023). Recent research has revealed attention-guided ensemble learning can improve intrusion detection capabilities by dynamically assigning context-aware priority to classifier outputs based on changing traffic characteristics (Hashmi et al., 2024; Singh et al., 2025).

In our previous work, the HAMC-ID framework introduced an attention-based meta-ensemble intrusion detection architecture that employed heterogeneous base learners and an attention-enhanced Bidirectional Long Short-Term Memory (BiLSTM) meta-classifier for adaptive decision fusion (Antony Joseph Raj and Madiajagan, 2025). While HAMC-ID significantly improved intrusion detection performance through dynamic ensemble aggregation, it did not incorporate explainable artificial intelligence (XAI) or secure trust validation mechanisms. While HAMC-ID demonstrated the effectiveness of attention-based meta-ensemble learning for intrusion detection, it did not address explainability, forensic transparency, or tamper-resistant validation. The proposed TAE-IDS substantially extends this architecture by integrating SHapley Additive exPlanations (SHAP)-based explainability and blockchain-assisted trust validation into a unified intrusion detection framework.

There are still a number of important problems that need to be resolved, even though the classification accuracy of current intelligent IDS frameworks is capable. Most intrusion detection algorithms continue to operate as opaque systems with complex prediction logic. Even when they achieve great detection performance, security experts often do not understand why a specific traffic instance is classified as benign or malicious. The implementation of intelligent IDS systems in real cybersecurity environments is limits deployment and reduces operational trust due to this absence of explainability. Furthermore, a lot of IDS frameworks ignore secure forensic auditing and trust verification in favor of predictive performance. Security archives and intrusion forecasts are typically reserved in centralized infrastructures that are vulnerable to unauthorized changes, manipulation, or tampering. Forensic analysis, intrusion tracing, and accountability are all vulnerable by the lack of safe and consistent logging systems.

To improve interpretability and transparency, intrusion detection systems have included recent explainable artificial intelligence (XAI) approaches with SHAP (Lundberg and Lee, 2017). The feature-level reasoning presented by SHAP-based intrusion detection frameworks helps analysts in understanding model behavior and detecting traits that motivate attacks (Ahmed et al., 2024). However, the majority of explainable IDS architectures use interpretability on their own without integrating tamper-proof validation or secure auditing. Similar to this, blockchain-based cybersecurity frameworks have recently come to light as viable decisions for secure intrusion tracking, immutable storage, and decentralized verification (Saveetha and Maragatham, 2022; Baig et al., 2023; Song et al., 2025; Pawar et al., 2025). Nevertheless, many of the recent blockchain-enabled IDS lack sophisticated explainable intrusion intelligence and adaptive ensemble learning capabilities, instead focused generally on secure storage or anomaly validation.

This paper suggests TAE-IDS, a Trust-Aware Explainable Intrusion Detection Framework utilizing Attention-Based Meta-Ensemble Learning with Blockchain Validation, as a solution to these issues. Within a single architecture, the suggested approach combines explainable AI, blockchain-secured intrusion auditing, and intelligent ensemble learning. XGB, ET, and Logistic Regression (LR) are used as heterogeneous base classifiers to capture a variety of network traffic characteristics. A BiLSTM attention-based meta-classifier is used to extract and fuse uncertainty-aware meta-features such as logits, confidence scores, and entropy representations to enhance adaptive ensemble aggregation. Additionally, the integration of SHAP-based explainability allows for the transparent reasoning of attack-driving features and model behavior by providing both global and local interpretation of intrusion predictions. A blockchain validation mechanism using SHA-256 hashing is integrated for tamper-proof storage and integrity verification of IDS predictions and security logs in order to improve credibility and forensic dependability.

The major contributions of this work are summarized as follows:

- Proposed an attention-based meta-ensemble intrusion detection framework integrating LR, ET and XGB.
- Incorporated uncertainty-aware meta-feature modeling using logits, confidence, and entropy.
- Developed a SHAP-based explainability framework for global and local intrusion reasoning.
- Designed a blockchain-inspired tamper-evident validation mechanism for tamper-proof intrusion logging.
- Extended the previously published HAMC-ID framework by integrating SHAP-based explainability and tamper-evident validation into a unified trust-aware intrusion detection architecture.
- Evaluated the proposed framework on the UNSW-NB15 and CICIDS2017 datasets under both binary and multiclass intrusion detection settings.

## 2 Related works

### 2.1 Ensemble-based intrusion detection

Research in intelligent intrusion detection systems that can correctly recognize changing attack behaviors has expanded dramatically due to the growing sophistication of cyber threats and the increasing heterogeneity of network settings. Due to their heavy reliance on pre-established rules and manually created attack signatures, traditional signature-based intrusion detection techniques frequently fail to identify dynamic intrusion patterns and 0-day attacks. Machine learning and deep learning-based IDS have been thoroughly investigated as a way to get around these restrictions because they can learn intricate traffic patterns and adaptive decision boundaries from network data (Ferrag et al., 2020). Among these creative approaches, ensemble learning methods have garnered a lot of interest because they boost classification resilience, decrease overfitting, and improve generalization capability by integrating many classifiers (Thockchom et al., 2023).

The application of ensemble learning for intrusion detection has been validated by numerous research. Thockchom et al. (2023) introduced an ensemble learning-based intrusion detection system (IDS) that incorporates multiple machine learning classifiers to improve detection stability under a range of network traffic circumstances. Their approach surpassed individual learners in categorization by utilizing the complementary strengths of several classifiers. In a similar vein, Farha and Ahmed (2024) presented a heterogeneous stacking-based intrusion detection architecture that aggregated multiple base learners via layered ensemble aggregation. Their method demonstrated improved attack detection and classification reliability on benchmark intrusion datasets.

Current advancements have gradually incorporated deep learning and attention processes into ensemble intrusion detection frameworks to improve adaptive decision fusion and context-aware intrusion classification. A hybrid feature-weighted attention-based deep learning system that integrated feature optimization and attention-guided learning for intrusion detection was offered by Hashmi et al. (2024). Their method boosted the accuracy of attack classification by dynamically weight discriminative network traffic characteristics throughout learning. Likewise, in order to increase temporal intrusion modeling and contextual decision fusion, Singh et al. (2025) presented an attentional LSTM-ensemble intrusion detection architecture for smart grid environments. In their framework, attention mechanisms dynamically reweighted sequential traffic representations. These experiments show how attention-guided ensemble designs can greatly enhance adaptive intrusion detection performance in dynamic and complex network environments.

Moreover, temporal intrusion detection frameworks based on deep learning have established a promising capability to model sequential network behaviors. For resource-constrained IoT contexts, Jouhari and Guizani (2024) suggested a lightweight CNN-BiLSTM intrusion detection framework that enhanced sequential traffic modeling and temporal feature extraction. Similar to this, current research has revealed that transformer and attention-driven intrusion detection frameworks are extremely capable of learning contextual traffic linkages and high-dimensional traffic dependencies (Umer et al., 2025). In spite of the fact that these frameworks significantly increased classification accuracy, their high computational complexity and poor interpretability often limit their use in real-time cybersecurity settings.

Adaptive learning techniques and feature optimization have been further examined in recent research to increase intrusion detection performance. For machine learning-based intrusion detection, Tripathi et al. (2024) proposed a weighted feature selection technique that uses adaptive weighting methods to increase feature relevance and classification accuracy. In the same way, a hybrid feature selection method for anomaly intrusion detection in IoT networks was presented by Ayad et al. (2024), improving detection capability through dimensionality reduction and optimal feature representation. These surveys exposed that adaptive feature intelligence plays an important role in enhancing classification stability and intrusion detection robustness.

Moreover, many ensemble-based intrusion detection frameworks have focused on enhancing minority-class detection capability and handling unbalanced attack distributions. That enhance classification performance on imbalanced intrusion datasets, Thakkar and Lohiya (2023) proposed an ensemble deep neural network framework for intrusion detection employing bagging-based learning techniques. Srinivasan and Deepalakshmi (2023) used well-organized feature engineering and ensemble aggregation to make an intrusion detection framework for botnet detection based on ensemble classification. These frameworks enhanced the ability to classify intrusions, but they did not include explainability procedures or trust-aware security validation, instead focused on predictive performance.

Even though ensemble and attention-based IDS have made great strides, there are still a number of important issues that need to be addressed. The majority of current frameworks prioritize detection accuracy while functioning as opaque, difficult-to-understand systems. In addition, a lot of ensemble designs rely on static aggregation techniques that are incapable to dynamically alter to shifting network behaviors and growing attack patterns. Furthermore, explainability, secure auditing, and trust verification processes are rarely permanently integrated into ensemble intrusion detection pipelines. Existing approaches typically handle feature optimization, adaptive learning, explainability, and trust validation as distinct components rather than integrating them into a single intrusion detection architecture.

Our previous HAMC-ID framework introduced an attention-based meta-ensemble architecture that dynamically aggregated heterogeneous base learners using an attention-enhanced BiLSTM meta-classifier. Although HAMC-ID significantly improved adaptive intrusion detection performance, it primarily focused on classification accuracy and adaptive ensemble learning. It did not address explainability or secure validation of intrusion records (Antony Joseph Raj and Madiajagan, 2025). These limitations motivate the proposed TAE-IDS framework, which integrates explainable AI and tamper-evident validation into a unified intrusion detection architecture.

### 2.2 Explainable intrusion detection

Attack detection capabilities have been greatly improved by the growing incorporation of AI and deep learning techniques into IDS; nonetheless, many intelligent IDS frameworks still function as opaque black-box systems with poor interpretability.

Recent research has used explainability techniques to provide clear reasoning and feature-level interpretation for intrusion detection results. First introduced by Lundberg and Lee (2017), SHapley Additive exPlanations (SHAP) has become one of the most widely used explainability frameworks due to its strong theoretical underpinnings and ability to provide both global and local model explanations. SHAP computes feature contribution scores using the ideas of cooperative game theory, allowing analysts to ascertain how each feature influences model predictions. SHAP-based interpretation enhances forensic analysis capabilities and aids security analysts in comprehending network features that drive attacks in intrusion detection systems.

Explainability approaches have been incorporated into intrusion detection systems in recent studies to increase analyst trust and transparency. For intrusion detection systems, Ahmed et al. (2024) suggested a hybrid bagging and boosting framework combined with SHAP-based feature interpretation. Their architecture made it possible to analyze intrusion-driving network parameters transparently and enhanced the interpretation of feature relevance. Similarly, recent explainable deep learning and ensemble architectures have shown that explainability techniques can greatly enhance comprehension of classifier behavior and help analysts more successfully spot anomalous traffic patterns (Singh et al., 2025).

Recurrent neural networks, BiLSTM models, and attention mechanisms are examples of deep learning architectures that have proven to be highly capable of learning complex temporal traffic dependencies and contextual linkages (Hashmi et al., 2024; Singh et al., 2025; Jouhari and Guizani, 2024). However, owing to their high-dimensional feature representations and non-linear decision boundaries, many of these models are still challenging to understand. Attention techniques frequently fail to give explicit feature-level reasoning for intrusion decisions, despite the fact that they partially increase interpretability by detecting contextually significant regions during prediction. As a result, deep learning systems are increasingly incorporating *post-hoc* explainability techniques like SHAP to offer transparent and interpretable intrusion reasoning.

Explainable intrusion detection has advanced recently, however there are still a number of issues. Without incorporating secure trust verification or tamper-proof auditing techniques, the majority of explainable IDS frameworks now in use concentrate mostly on feature interpretation. Rather of being integrated into a single, trust-aware intrusion detection architecture, explainability modules are frequently handled as separate post-processing elements. Additionally, a number of explainable intrusion detection systems lack localized reasoning for specific intrusion predictions and instead offer global interpretation. For real-time intrusion detection situations, explainability approaches' computing overhead continues to be a problem.

Adaptive ensemble learning and attention-based aggregation were the main focuses of HAMC-ID in our earlier research to enhance intrusion detection performance (Antony Joseph Raj and Madiajagan, 2025). Although HAMC-ID improved contextual decision fusion and classification accuracy, it did not provide mechanisms to explain individual intrusion decisions or to support analyst interpretation. The proposed TAE-IDS addresses this limitation by integrating SHAP-based explainability to provide both global and local explanations while preserving adaptive ensemble learning.

### 2.3 Blockchain-based security frameworks

Blockchain technology has been included into intrusion detection and cybersecurity frameworks due to the growing sophistication of cyber-attacks and the increasing dependence on distributed digital infrastructures. Blockchain is a potential solution for safeguarding IDS and network security infrastructures since it offers decentralized verification, immutable data storage, cryptographic integrity protection, and tamper-resistant auditing. So as to improve trustworthiness, secure log management, and cooperative threat intelligence sharing across distributed contexts, a growing number of recent studies have explored blockchain-enabled intrusion detection frameworks (Saveetha and Maragatham, 2022; Baig et al., 2023; Song et al., 2025; Pawar et al., 2025).

Several studies have combined blockchain technology with deep learning and machine learning methods to enhance secure network monitoring and intrusion detection accuracy. For secure data storage and intrusion monitoring, Saveetha and Maragatham (2022) suggested a blockchain-enabled deep learning intrusion detection system. Their methodology lacked explainability integration and adaptive feature optimization, but it did increase intrusion detection capability and security reliability Similarly, Baig et al. (2023) presented a blockchain and stacking machine learning architecture for detecting rogue nodes in Internet of Things scenarios that is connected with InterPlanetary File System (IPFS) storage. Their method enhanced secure distributed intrusion monitoring, but it lacked sophisticated attention-guided learning methods and depended on somewhat basic classifiers.

Deep learning and temporal modeling techniques for secure cyberattack detection have been actively investigated in recent blockchain-enabled intrusion detection systems. For safe healthcare IoT connectivity, Kumar et al. (2023) suggested a blockchain-orchestrated intrusion detection system that combines DSAE, BiLSTM, blockchain, IPFS, and zero-knowledge proof techniques. Although their framework enhanced intrusion detection and safe data transmission, real-time scalability was constrained by the high processing complexity. Furthermore, a blockchain-enhanced deep learning intrusion detection system using LSTM-based learning and secure execution environments for adaptive intrusion monitoring was presented by Aliyu et al. (2025). The framework demonstrated significant architectural complexity and restricted feature engineering capacity, despite improving adaptability and secure execution.

For distributed and Internet of Things contexts, a number of lightweight and optimized blockchain-enabled intrusion detection solutions have also been developed. For DDoS detection in IoT networks, Kumari et al. (2024) combined blockchain-secured malicious IP logging with Random Forest categorization. Although the architecture improved secure attack recording, it was limited to DDoS-oriented scenarios and lacked advanced ensemble learning and explainability features. Additionally, Ismail et al. (2024) proposed a lightweight blockchain-based intrusion detection system utilizing LightGBM and smart contracts for trust-aware intrusion monitoring in wireless sensor networks. Their method lacked advanced deep learning architectures and attention approaches, although having a lower computational overhead.

Recent research has also examined the integration of explainable AI, feature optimization, and attention processes into blockchain-enabled intrusion detection systems. Song et al. (2025) introduced a hybrid blockchain and machine learning intrusion detection technique that leverages XGB and blockchain-inspired tamper-evident validation for industrial IoT scenarios. Although their approach reduced false positives and improved intrusion monitoring reliability, it relied on a single classifier without adaptive ensemble diversity or attention-guided aggregation. Pawar et al. developed Cyber-Shield, a blockchain-protected intrusion detection system that integrates ensemble learning and optimization techniques for safe intrusion logging and network monitoring (Pawar et al., 2025). Its system improved security auditing and classification performance even though it lacked deep learning architectures and contextual attention approaches. By incorporating machine learning, blockchain, and SHAP-based explainability into an IoT intrusion detection system, Kumar et al. (2025) enhanced interpretability and trust-aware security analysis. The system lacked advanced meta-feature modeling and adaptive attention-based ensemble fusion, despite an improvement in explainability.

More recent research has focused on incorporating blockchain-inspired tamper-evident validation and attention techniques into intrusion detection pipelines. Sunanda et al. (2024) combined feature optimization, Attention-BiLSTM structures, and blockchain-inspired tamper-evident validation for IoT intrusion detection. Their framework's capacity to improve sequential traffic learning and detection accuracy was hampered by its high computational complexity and low interpretability. Similarly, Alharthi et al. (2024) proposed federated learning and a blockchain-based anomaly detection system for IoT security that preserves anonymity. Despite enhancing distributed trust and privacy protection, the system had a significant communication overhead and no capacity for explainable intrusion reasoning.

Despite recent advancements, blockchain-enabled intrusion detection systems still have a number of serious problems. Most existing solutions primarily focus on distributed validation, secure storage, or anomaly detection without integrating explainable AI and adaptive ensemble learning into a single architecture. Additionally, because many blockchain-based intrusion detection systems rely on static learning algorithms or single classifiers, their ability to adapt to shifting attack patterns and a variety of network behaviors is limited. Furthermore, explainability, trust verification, adaptive ensemble aggregation, and secure intrusion auditing are often treated as distinct processes rather being integrated into a comprehensive trust-aware intrusion detection architecture.

Our previous HAMC-ID framework demonstrated that attention-guided ensemble learning can improve intrusion detection performance. However, it lacked secure validation mechanisms and explainability. The proposed TAE-IDS addresses these limitations by integrating blockchain-assisted tamper-evident validation and SHAP-based explainability into a unified trust-aware intrusion detection framework.

Table 1 summarizes recent intrusion detection frameworks that integrate ensemble learning, explainable artificial intelligence, deep learning, attention mechanisms, and blockchain-enabled security validation. The comparison highlights the strengths and limitations of existing approaches in terms of explainability, adaptive ensemble learning, trust verification, and secure intrusion auditing.

**Table 1:** Summary of existing IDS models and limitations.

Author	Technique/classifier	Key contribution	Dataset/environment	Limitations
Saveetha and Maragatham (2022)	Deep learning + blockchain IDS	Integrates blockchain for secure data storage with DL-based intrusion detection	General network IDS	No feature optimization or explainability; limited scalability analysis
Baig et al. (2023) (BEML)	Stacking ensemble (LDA, DT, perceptron) + blockchain + IPFS	Combines blockchain with stacked ML for secure IoT attack detection	IoT environment	Uses basic classifiers; lacks deep learning and attention mechanisms
Kumar et al. (2023)	DSAE + BiLSTM + blockchain + IPFS + ZKP	Secure data transmission + IDS with deep learning and blockchain orchestration	CICIDS2017, ToN-IoT (healthcare IoT)	High computational complexity; lacks explainability
Thakkar and Lohiya (2023)	Bagging-based deep neural network	Handles class imbalance using ensemble DNN for IDS	NSL-KDD, UNSW-NB15, CICIDS2017, BoT-IoT	No security layer (blockchain); lacks interpretability
Srinivasan and Deepalakshmi (2023)	Stacking ensemble (ECASP)	Detects botnets using ensemble learning and feature optimization	Botnet datasets	Limited generalization; no deep learning or secure framework
Venkatesan and Rahayu (2024)	Hybrid consensus + ML	Enhances blockchain security using ML-based consensus optimization	Blockchain platforms (ProximaX)	Focuses on blockchain layer only; no IDS integration
Kumari et al. (2024)	Random forest + blockchain logging	Stores malicious IPs securely in blockchain for DDoS detection	CICIDS2017, CICDoS2016	Limited to DDoS; lacks advanced learning models
Ismail et al. (2024)	LightGBM + blockchain (smart contracts)	Lightweight security framework with trust + detection modules	WSN-DS dataset (IoT)	Limited model complexity; lacks attention and explainability
Aliyu et al. (2025)	LSTM + blockchain + TEE	Self-adaptive IDS with continuous learning and secure execution	Blockchain platforms	High system complexity; limited feature engineering
Song et al. (2025)	XGB + blockchain IDS	Reduces false positives in IIoT using ML + blockchain integration	BOT-IoT dataset	Single classifier; lacks ensemble diversity
Pawar et al. (2025) (cyber-shield)	Ensemble ML + blockchain + ACO	Secure log storage + improved accuracy using optimization	NSL-KDD, CICIDS2017	No deep learning or attention mechanisms
Kumar et al. (2025) (BCE-IoT)	ML + blockchain + SHAP	Combines explainable AI with blockchain for secure IDS	IoT datasets	Limited ensemble modeling; lacks attention-based fusion
Alharthi et al. (2024)	Federated learning + blockchain	Privacy-preserving distributed IDS using FL	IoT environments	High communication overhead; lacks explainability
Hashmi et al. (2024)	CNN + BiLSTM + attention + RF	Feature-weighted attention for improved IDS accuracy	NSL-KDD, UNSW-NB15	No security layer (blockchain); high complexity
Ahmed et al. (2024)	SHAP + hybrid bagging-boosting	Explainable feature selection improves IDS performance	Standard IDS datasets	No security framework; lacks temporal modeling
Umer et al. (2025)	Transformer (multi-head attention)	Improves IDS accuracy using transformer-based architecture	UNSW-NB15	High computational cost; no blockchain or explainability
Singh et al. (2025)	Attention + LSTM + ensemble (XGB, LGBM, CatBoost)	Combines temporal learning with ensemble for IDS	Smart grid dataset	Moderate accuracy; lacks security and explainability
Kumar et al. (2022) (JPDC)	Random forest + XGB (fog-based distributed IDS + blockchain)	Distributed IDS for DDoS detection in blockchain-enabled IoT networks	BoT-IoT dataset	Limited to DDoS; lacks ensemble fusion, attention, and explainability
Nouman et al. (2023)	Histogram gradient boost + blockchain + IPFS + VBFT	Detects malicious nodes and ensures secure storage using blockchain	WSN-DS dataset	Single model; lacks ensemble learning, attention, and explainability
Khonde and Ulagamuthalvi (2022)	Hybrid IDS + blockchain (BC-HyIDS)	Secures signature sharing in distributed IDS using blockchain	Distributed IDS environment	Limited ML sophistication; lacks deep learning and adaptive modeling
Jemili and Korbaa (2023)	Collaborative IDS + blockchain + ML	Enables decentralized IDS with collaborative learning	Distributed network environment	Lacks advanced deep learning, attention, and explainability
Sunanda et al. (2024) (IJACSA)	RFO + attention BiLSTM + blockchain	Improves IDS accuracy using feature optimization and attention with blockchain	IoT datasets	High complexity; limited generalization and interpretability
Antony Joseph Raj and Madiajagan (2025) (HAMC-ID)	Attention-based meta-ensemble (GNB, LR, XGB + BiLSTM attention)	Introduces meta-feature driven attention-based ensemble for intrusion detection	UNSW-NB15, CICIDS2017	Lacks secure logging mechanism and explainability integration

#### 2.3.1 Research gaps

- Existing IDS frameworks primarily focus on detection accuracy while lacking integrated explainability and trust verification mechanisms.
- Most blockchain-inspired tamper-evident validation layer models emphasize secure storage or anomaly validation without adaptive ensemble intelligence and explainable reasoning.
- Current explainable IDS frameworks provide limited integration with tamper-proof auditing and blockchain-secured intrusion validation.
- No unified trust-aware intrusion detection framework effectively integrates attention-based ensemble learning, explainable AI, and blockchain-secured auditing within a single architecture.

As shown in Table 2, the proposed TAE-IDS extends the previously published HAMC-ID framework by incorporating explainability and trust-aware validation capabilities while preserving the attention-based heterogeneous ensemble architecture. These additions improve transparency, auditability, and operational trust without altering the fundamental adaptive ensemble learning strategy.

**Table 2:** Architectural comparison between HAMC-ID and the proposed TAE-IDS.

Component	HAMC-ID	TAE-IDS (proposed)
Heterogeneous ensemble	✓	✓
Attention BiLSTM	✓	✓
Meta-feature learning	✓	✓
SHAP explainability	✗	✓
Global explanations	✗	✓
Local explanations	✗	✓
Tamper-evident validation	✗	✓
Trust-aware IDS	✗	✓
Secure forensic logging	✗	✓

## 3 Proposed model

### 3.1 System overview

This work proposes TAE-IDS, a Trust-Aware Explainable Intrusion Detection Framework that combines blockchain-inspired tamper-evident validation, explainable artificial intelligence, and attention-based meta-ensemble learning into a single architecture to address the shortcomings of current intrusion detection systems in terms of transparency, trustworthiness, and secure decision validation. The proposed model simultaneously stresses adaptive intrusion detection, interpretable decision-making, and tamper-proof preservation of intrusion records, in contrast to traditional IDS frameworks that prioritize increasing detection accuracy. The suggested architecture is tested on a variety of network intrusion datasets and covers both binary and multiclass intrusion detection scenarios.

**Figure 1:**
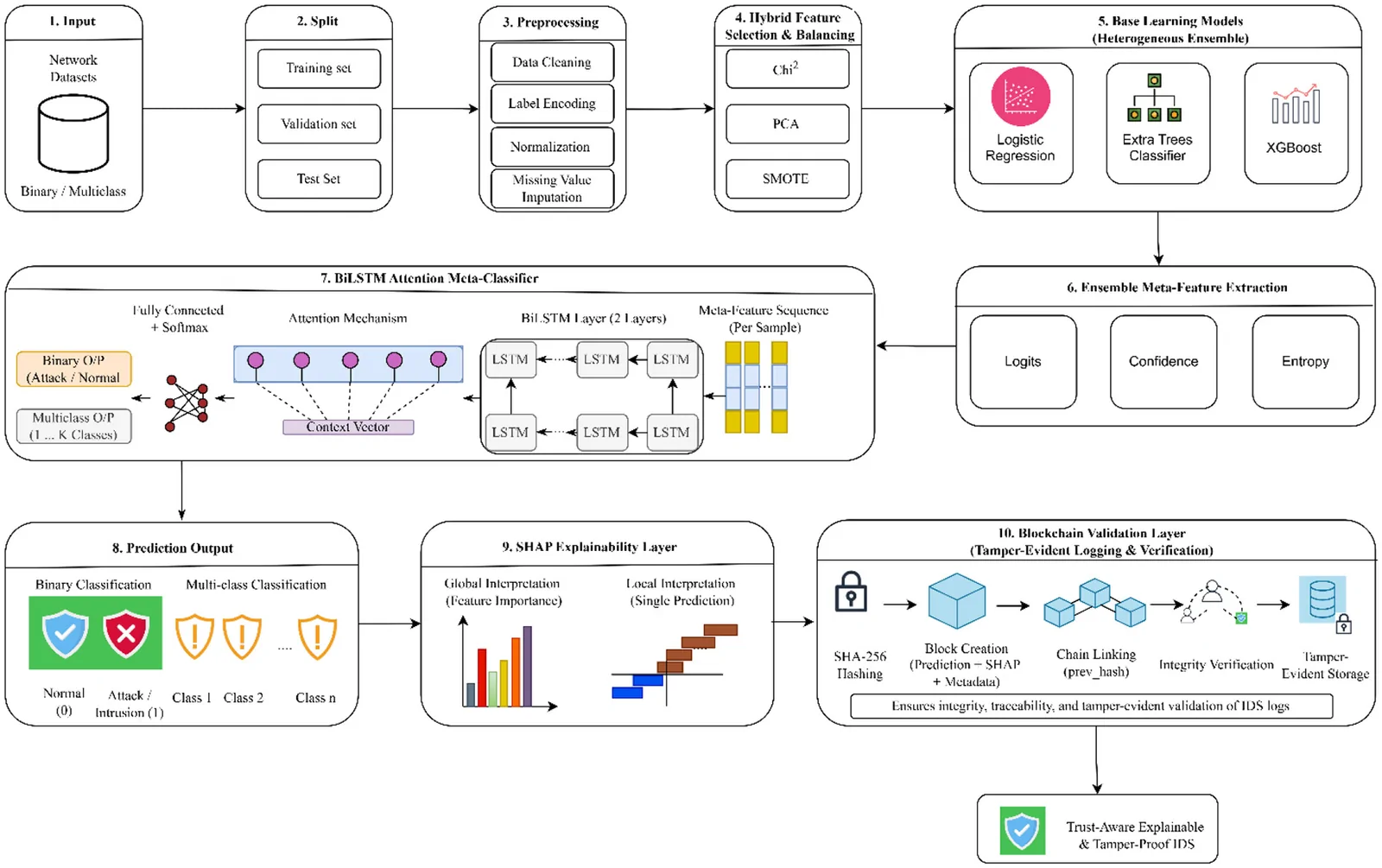
TAE-IDS framework.

The general design of the suggested TAE-IDS framework is shown in Figure 1. Let the intrusion detection dataset be represented as:


$$\begin{array}{c} D=\left\{X,Y\right\} \end{array}$$

where *X* = {*x*_1_, *x*_2_, …, *x*_*n*_} denotes the network traffic feature space and *Y* = {*y*_1_, *y*_2_, …, *y*_*n*_} represents the corresponding intrusion labels for binary or multiclass attack categories. Network traffic data from benchmark network security datasets is the first step in the intrusion detection pipeline. To provide trustworthy model optimization and objective performance assessment, the datasets are divided into training, validation, and testing subsets. To enhance feature consistency and learning stability over various traffic distributions, the preparation step includes data cleaning, label encoding, missing value imputation, and Min–Max normalization.

After preprocessing, a hybrid feature selection and balancing stage is applied to the improved traffic features. Principal Component Analysis (PCA) is used to reduce feature dimensionality and remove redundant correlations, while Chi-Square (χ^2^) feature selection is used to find statistically significant characteristics linked to intrusion behavior. The Class imbalance problems that are commonly observed in intrusion detection datasets are addressed by using the Synthetic Minority Oversampling Technique (SMOTE) (Chawla et al., 2002) to provide balanced training samples and improve minority attack recognition skills.

To prevent information leakage, all preprocessing operations were performed exclusively using the training data. Specifically, Min–Max normalization parameters were estimated from the training set and subsequently applied to the validation and test sets using the same transformation. Similarly, Chi-square feature selection and Principal Component Analysis (PCA) were fitted only on the training data before transforming the validation and test data. SMOTE oversampling was applied exclusively to the training partition after feature transformation and was never applied to the validation or test sets. Consequently, no information from the validation or testing data was used during model training or feature engineering, ensuring an unbiased evaluation of the proposed framework.

The balanced and optimized feature space is then provided to the heterogeneous ensemble base learners, which comprise XGB (Chen and Guestrin, 2016), ET (Geurts et al., 2006), and LR. These classifiers were selected because of their complimentary learning properties and computational efficiency. XGB promotes gradient-based optimization and complex feature interaction modeling, ET improves non-linear ensemble-based pattern learning, and LR offers robust linear decision bounds. For intrusion classification, each classifier independently learns a mapping function:


$$\begin{array}{c} f_{m}:X\to Ŷ,m\in \left\{LR,ET,XGB\right\} \end{array}$$

where Ŷ denotes the predicted intrusion outputs generated by the corresponding base classifiers.

Instead of immediately aggregating base classifier predictions using static fusion techniques, the proposed model obtains structured ensemble meta-features from the base models' outputs. Among these meta-features are entropy values, logits, and prediction confidence ratings. Logits preserve the raw classifier decision information prior to probability normalization, entropy values show ambiguity regarding classifier outputs, and confidence scores show prediction certainty. When combined, these meta-features provide more specific contextual information about prediction accuracy and classifier behavior. For every traffic sample, the produced meta-feature representation can be written as follows:


$$\begin{array}{c} \phi_{i}=\left[l_{i},c_{i},e_{i}\right] \end{array}$$

where *l*_*i*_, *c*_*i*_, and *e*_*i*_ denote the logit, confidence, and entropy values associated with sample *i*, respectively.

An attention-enhanced BiLSTM meta-classifier then processes the resulting meta-feature sequence. Sequential representation learning across classifier outputs is made possible by the BiLSTM layers, which capture forward and backward contextual dependencies across ensemble meta-features. The attention mechanism then prioritizes trustworthy classifier contributions during intrusion prediction by dynamically allocating adaptive relevance weights to useful meta-features. In order to generate binary and multiclass intrusion detection outputs, the weighted contextual representation is ultimately processed through fully connected and softmax layers. The suggested attention-guided meta-learning approach enhances contextual decision fusion, adaptive generalization capacity, and robustness against intricate intrusion patterns when compared to traditional static stacking ensembles (Antony Joseph Raj and Madiajagan, 2025).

The proposed model incorporates a SHAP-based explainability layer after the prediction stage to enhance interpretability and analyst trust. Both local and global interpretation of intrusion decisions are provided by the explainability module. While local explanations offer instance-level justification for specific intrusion predictions, global explanations pinpoint the most significant meta-features influencing the framework's overall behavior. Security analysts may better understand classifier behavior, look into aspects that lead to attacks, and boost trust in automated IDS choices thanks to its transparent reasoning capabilities (Lundberg and Lee, 2017).

Finally, TAE-IDS incorporates a blockchain-inspired tamper-evident validation layer to ensure tamper-proof preservation and trustworthy verification of intrusion detection outputs. The validation layer performs SHA-256 hashing, secure block creation, previous-hash chain linking, and integrity verification of intrusion records using a tamper-evident hash-chain. Prediction outputs and associated explainability metadata are stored in each block to provide integrity verification, traceability, and non-repudiation of IDS decisions. Unlike traditional IDS logging systems, which are still vulnerable to tampering or deletion, the blockchain-inspired tamper-evident validation technique ensures that intrusion records cannot be altered without discovery (Saveetha and Maragatham, 2022; Baig et al., 2023).

The proposed TAE-IDS framework integrates explainable artificial intelligence, blockchain-inspired tamper-evident validation, and adaptive ensemble learning into a single architecture to provide an effective and dependable intrusion detection solution that can support transparent, interpretable, and secure cybersecurity monitoring environments.

### 3.2 Base detection model

#### 3.2.1 Heterogeneous base learners

A heterogeneous ensemble-based intrusion detection architecture with LR, ET (Geurts et al., 2006), and XGB (Chen and Guestrin, 2016) as Level-0 base learners is used in the suggested TAE-IDS framework. Let the optimized feature space obtained after preprocessing and feature refinement be represented as:


$$\begin{array}{c} F^{*}\in {\mathbb{R}}^{n\times d} \end{array}$$

where *n* denotes the number of traffic samples and *d* represents the number of selected features. Each base learner independently learns a mapping function:


$$\begin{array}{c} f_{m}:F^{*}\to Ŷ,m\in \left\{LR,ET,XGB\right\} \end{array}$$

where Ŷ denotes the predicted intrusion probabilities.

LR is incorporated as a lightweight discriminative classifier that estimates the conditional probability of intrusion using the sigmoid activation function:


$$\begin{array}{c} P\left(y=1|x\right)=\frac{1}{1+e^{-\left(w^{T}x+b\right)}} \end{array}$$ (1)

where *w* ∈ ℝ^*d*^ represents the learned weight vector, *b* ∈ ℝ denotes the bias term, and *x* ∈ ℝ^*d*^ is the input feature vector. The model parameters are optimized by minimizing the binary cross-entropy loss (Equation 1–8):


$$\begin{array}{c} L_{LR}=-\frac{1}{n}\sum_{i=1}^{n}\left[y_{i}\log\left({ŷ}_{i}\right)+\left(1-y_{i}\right)\log\left(1-{ŷ}_{i}\right)\right] \end{array}$$ (2)

where *y*_*i*_ and ŷ_*i*_denote the actual and predicted labels, respectively.

The ET classifier is a randomized ensemble learning model that improves classification diversity through random feature selection and randomized split generation. Let *f*_*k*_(*x*) denotes the prediction of the *k*th decision tree. The final ensemble prediction is obtained using majority voting:


$$\begin{array}{c} F\left(x\right)=\mathrm{mode}\left(f_{1}\left(x\right),f_{2}\left(x\right),\ldots ,f_{K}\left(x\right)\right) \end{array}$$ (3)

where *K* denotes the total number of trees in the ensemble. The randomized structure of ET reduces variance and improves robustness against noisy and heterogeneous network traffic distributions.

XGB is incorporated as a gradient-boosting framework capable of learning non-linear feature interactions and adaptive decision boundaries. At iteration *t*, the objective function is formulated as:


$$\begin{array}{c} L^{\left(t\right)}=\sum_{i=1}^{n}l\left(y_{i},{ŷ}_{i}^{\left(t\right)}\right)+\sum_{k=1}^{t}\Omega \left(f_{k}\right) \end{array}$$ (4)

where $l\left(y_{i},{ŷ}_{i}^{\left(t\right)}\right)$ represents the prediction loss and Ω(*f*_*k*_) denotes the regularization term controlling model complexity:


$$\begin{array}{c} \Omega \left(f\right)=\gamma T+\frac{1}{2}\lambda \sum_{j=1}^{T}w_{j}^{2} \end{array}$$ (5)

where *T* is the number of leaves, *w*_*j*_ denotes the leaf weights, γ penalizes excessive tree growth, and λ controls L2 regularization. XGB effectively captures high-dimensional non-linear intrusion relationships while maintaining strong generalization capability.

#### 3.2.2 Meta-feature construction

Unlike conventional stacking approaches that directly combine prediction labels or probabilities, TAE-IDS extracts structured uncertainty-aware meta-features from each base learner. For each traffic sample *x*_*i*_ and each base classifier *m*, three categories of meta-features are extracted.

The logit score preserves raw classifier decision information prior to probability normalization:


$$\begin{array}{c} l_{i}^{m}=\log\left(\frac{p_{i}^{m}}{1-p_{i}^{m}}\right) \end{array}$$ (6)

where $p_{i}^{m}\;$denotes the predicted probability generated by classifier *m*.

Classifier confidence is computed as the maximum predicted class probability:


$$\begin{array}{c} c_{i}^{m}=\max_{k}\left(p_{i,k}^{m}\right) \end{array}$$ (7)

where $p_{i,k}^{m}\;$represents the probability of class *k*.

Prediction uncertainty is quantified using entropy:


$$\begin{array}{c} e_{i}^{m}=-\sum_{k}p_{i,k}^{m}\log\left(p_{i,k}^{m}\right) \end{array}$$ (8)

Higher entropy values indicate increased uncertainty in classifier predictions.

The meta-feature vector for sample *i* is represented as:


$$\begin{array}{c} \phi_{i}=\left[l_{i}^{LR},c_{i}^{LR},e_{i}^{LR},l_{i}^{ET},c_{i}^{ET},e_{i}^{ET},l_{i}^{XGB},c_{i}^{XGB},e_{i}^{XGB}\right] \end{array}$$

The generated meta-feature tensor is defined as:


$$\begin{array}{c} \text{Φ}\in {\mathbb{R}}^{n\times T\times f} \end{array}$$

where *T* = 3 represents the number of base classifiers and *f* = 3 denotes the number of meta-features extracted from each classifier.

Contextual information about classifier confidence, reliability, and prediction ambiguity is provided by these uncertainty-aware meta-features. Additionally, the explainability module's SHAP-based intrusion reasoning is supported and interpretability is enhanced by the extracted meta-representations.

#### 3.2.3 Attention-based BiLSTM meta-classifier

An attention-enhanced BiLSTM meta-classifier is used to process the resulting meta-feature sequence (Equations 9–18). Forward and backward contextual relationships between ensemble meta-features are learned by the BiLSTM network:


$$\begin{array}{c} h_{t}=\mathrm{BiLSTM}\left(\phi_{t};\theta \right) \end{array}$$ (9)

where *h*_*t*_ represents the hidden representation at timestep *t*, and θ denotes the BiLSTM parameters.

To adaptively prioritize reliable classifier outputs, an attention mechanism computes contextual importance weights:


$$\begin{array}{c} \alpha_{t}=\frac{\exp\left(w_{a}^{T}\tanh\left(W_{h}h_{t}\right)\right)}{\sum_{j=1}^{T}\exp\left(w_{a}^{T}\tanh\left(W_{h}h_{j}\right)\right)} \end{array}$$ (10)

where α_*t*_ denotes the attention weight assigned to the hidden state *h*_*t*_.

The final context vector is computed as:


$$\begin{array}{c} c=\sum_{t=1}^{T}\alpha_{t}h_{t} \end{array}$$ (11)

The weighted contextual representation is then forwarded to fully connected and softmax layers for final intrusion prediction:


$$\begin{array}{c} ŷ=\mathrm{softmax}\left(W_{c}c\right) \end{array}$$ (12)

In contrast to static ensemble fusion techniques, the proposed attention-guided meta-learning approach suppresses ambiguous or less informative outputs while dynamically emphasizing trustworthy classifier results. Although the uncertainty-aware meta-feature sequence contains only a small number of heterogeneous classifier representations, the BiLSTM is employed to model contextual dependencies among classifier outputs rather than long temporal sequences. Each sequence element represents the uncertainty-aware representation of an individual base learner, allowing the bidirectional architecture to capture complementary relationships before the attention mechanism performs adaptive weighting. Consequently, the BiLSTM serves primarily as a contextual feature fusion module rather than a conventional long-sequence temporal model, enabling adaptive integration of complementary classifier representations before the attention mechanism performs the final aggregation. This adaptive aggregation mechanism improves contextual intrusion reasoning, enhances minority-class sensitivity, and increases robustness against evolving cyberattack patterns across both binary and multiclass intrusion detection environments.

### 3.3 Explainability module (SHAP)

Despite the high detection performance achieved by deep learning and ensemble-based intrusion detection systems, many intelligent IDS frameworks still operate as opaque black-box models. In cybersecurity environments, predictions without interpretable reasoning reduce analyst confidence and complicate forensic investigation. Security analysts require explanations that identify why a network flow is classified as malicious, which traffic characteristics contribute most strongly to the prediction, and how classifier uncertainty influences the final intrusion decision. To address these challenges, the proposed TAE-IDS framework incorporates a SHAP-based explainability module that provides both global and local interpretations of intrusion predictions.

The explainability layer is applied after the attention-based BiLSTM meta-classification stage. The proposed framework adopts a two-level explainability strategy. At the first level, SHAP is applied to the uncertainty-aware ensemble meta-features generated by the heterogeneous base learners to explain the decision-making process of the attention-based meta-classifier. At the second level, TreeSHAP is applied to the most influential base learner, identified from the first-level SHAP analysis, to determine the contribution of the original network traffic features to intrusion detection. This complementary strategy provides both ensemble-level transparency and traffic-level attribution. The uncertainty-aware meta-features consist of the logits, confidence scores, and entropy values generated by the Logistic Regression (LR) and Extra Trees (ET) base learners, which are used as inputs to the attention-based meta-classifier. By analyzing these meta-representations, the explainability module provides interpretable reasoning regarding classifier reliability, uncertainty behavior, and contextual decision contribution within the ensemble architecture.

The proposed model calculates feature contribution scores for intrusion predictions using SHAP (Lundberg and Lee, 2017). Based on cooperative game theory, SHAP computes Shapley values to estimate each feature's contribution to the final model prediction. Let the prediction function of the meta-classifier be represented as:


$$\begin{array}{c} f\left(\phi_{i}\right) \end{array}$$

where ϕ_*i*_ denotes the meta-feature vector associated with traffic sample *i*. The SHAP explanation model approximates the prediction function as:


$$\begin{array}{c} f\left(\phi_{i}\right)=\phi_{0}+\sum_{j=1}^{M}\phi_{j} \end{array}$$ (13)

where ϕ_0_ represents the baseline prediction and ϕ_*j*_ denotes the SHAP contribution of the *j*th meta-feature to the final intrusion prediction.

The Shapley value associated with each feature is computed as:


$$\begin{array}{c} \phi_{j}=\sum_{S\subseteq F\backslash \left\{j\right\}}\frac{|S|!\left(|F|-|S|-1\right)!}{|F|!}\left[f\left(S\cup \left\{j\right\}\right)-f\left(S\right)\right] \end{array}$$ (14)

where *F* denotes the complete feature set and *S* represents a subset of features excluding feature *j*. This formulation measures the marginal contribution of each feature across all possible feature combinations.

Within the proposed TAE-IDS framework, the first level of SHAP analysis is applied to the uncertainty-aware meta-feature space:


$$\begin{array}{c} \phi_{i}=\left[l_{i}^{LR},c_{i}^{LR},e_{i}^{LR},l_{i}^{ET},c_{i}^{ET},e_{i}^{ET}\right] \end{array}$$

where *l*_*i*_, *c*_*i*_, and *e*_*i*_ denote the logit, confidence, and entropy values generated by the corresponding base learner. This first-level analysis explains how the heterogeneous ensemble contributes to the final decision of the attention-based meta-classifier. Subsequently, TreeSHAP is applied to the most influential base learner identified from the first-level SHAP analysis to determine the original network traffic features responsible for individual intrusion predictions.

The explainability module generates both global and local interpretability outputs. Global SHAP analysis quantifies the overall importance of the uncertainty-aware meta-features across the intrusion detection framework. The global importance of feature *j* is computed using the mean absolute SHAP value as:


$$\begin{array}{c} I_{j}=\frac{1}{N}\sum_{i=1}^{N}|\phi_{j}^{\left(i\right)}| \end{array}$$ (15)

where *N* denotes the total number of traffic samples. Global interpretation enables analysts to identify which classifier outputs and uncertainty-aware representations most strongly influence the ensemble decision. The global SHAP importance obtained from the first-level analysis is subsequently used to identify the most influential base learner for the second-level TreeSHAP analysis. Local SHAP explanations provide instance-level reasoning by quantifying the positive and negative contributions of individual features to each intrusion prediction, thereby enabling transparent interpretation of the model's decision-making process. Together, the two levels of SHAP analysis provide complementary explanations by revealing both how the ensemble reaches its final decision and which original network traffic features are primarily responsible for the predicted intrusion.

### 3.4 Blockchain validation mechanism

Even though modern intrusion detection systems have great categorization capabilities, conventional IDS recording techniques are nevertheless vulnerable to unauthorized alteration, deletion, and manipulation of intrusion reports. In distributed cybersecurity systems, compromised logging infrastructure can allow attackers to secretly alter security data, compromise forensic investigation, and erode trust in automated intrusion judgments. The suggested TAE-IDS system includes a blockchain-inspired tamper-evident validation module that allows for safe auditing, tamper-proof preservation, and reliable verification of intrusion detection outputs in order to overcome these drawbacks.

The TAE-IDS framework's explainability stage is followed by the integration of the blockchain layer. After the intrusion prediction and SHAP-based explanation stages have been completed, the resulting prediction outputs and explainability metadata are forwarded to the blockchain validation layer for post-inference logging, SHA-256 hash generation, chain linking, and integrity verification. The blockchain validation module operates exclusively after model inference and does not participate in model training or parameter optimization. In both binary and multiclass intrusion detection environments, this decentralized validation method guarantees integrity preservation, traceability, and non-repudiation of IDS conclusions.

#### 3.4.1 Validation architecture

The proposed validation layer is implemented as a lightweight blockchain-inspired tamper-evident hash-chain. Each IDS prediction generates one block containing prediction metadata and SHAP explanations. Blocks are linked using SHA-256 hashes to ensure integrity. The current implementation operates as a single-node validation mechanism and does not employ distributed consensus protocols such as Proof-of-Authority (PoA), Practical Byzantine Fault Tolerance (PBFT), or Proof-of-Work (PoW). Therefore, the validation layer focuses on integrity preservation and forensic traceability rather than decentralized consensus.

The blockchain validation layer was implemented as a lightweight custom Python-based private permissioned blockchain operating on a single validation node. Unlike public blockchain platforms such as Ethereum or Hyperledger Fabric, the proposed implementation is specifically designed for tamper-evident forensic logging of IDS outputs rather than decentralized transaction processing. Since the objective of this work is integrity verification of intrusion detection records, consensus protocols such as Proof-of-Work (PoW), Proof-of-Authority (PoA), and Practical Byzantine Fault Tolerance (PBFT) were not employed. Instead, blockchain integrity is maintained through SHA-256 cryptographic hashing and previous-hash chain linking. Each intrusion prediction generates one blockchain block containing prediction metadata, SHAP explanations, timestamps, previous hash values, and cryptographic hash values. The proposed validation architecture provides lightweight secure forensic logging with minimal computational overhead while preserving tamper-evident integrity.

#### 3.4.2 Block structure

Each blockchain block stores intrusion-related information generated by the TAE-IDS framework. Let the *i*th blockchain block be represented as:


$$\begin{array}{c} B_{i}=\left\{ID_{i},T_{i},P_{i},S_{i},H_{i-1},H_{i}\right\} \end{array}$$

where *ID*_*i*_ denotes the block identifier, *T*_*i*_ represents the timestamp, *P*_*i*_ denotes the intrusion prediction output, *S*_*i*_ represents SHAP-based explainability information, *H*_*i*−1_ denotes the hash of the previous block, *H*_*i*_ represents the current block hash. Each block stores approximately 300–600 bytes depending on the size of SHAP metadata.

The stored prediction outputs include binary or multiclass intrusion labels together with prediction confidence and explainability metadata generated by the SHAP module.

#### 3.4.3 SHA-256 hashing mechanism

To ensure tamper resistance, each blockchain block is protected using the Secure Hash Algorithm SHA-256. The hash value of block *B*_*i*_ is computed as:


$$\begin{array}{c} H_{i}=SHA256\left(ID_{i}∥T_{i}∥P_{i}∥S_{i}∥H_{i-1}\right) \end{array}$$ (16)

where ∥ denotes concatenation of block attributes. The SHA-256 hashing operation generates a fixed-length cryptographic hash that uniquely represents the content of the block.

Any modification to intrusion records, prediction outputs, or explainability metadata changes the resulting hash value, thereby enabling immediate detection of unauthorized alterations.

#### 3.4.4 Chain linking and secure logging

The blockchain validation layer maintains sequential linkage between blocks using previous-hash references. For consecutive blocks *B*_*i*_ and *B*_*i*+1_, the chain relationship is defined as:


$$\begin{array}{c} H_{i}^{current}=H_{i+1}^{previous} \end{array}$$ (17)

This chained architecture ensures that each block depends on the integrity of all preceding blocks. Consequently, tampering with a single intrusion record invalidates all subsequent blockchain links.

After classification, the TAE-IDS framework securely logs the following information into blockchain blocks: predicted intrusion labels, confidence scores, SHAP explanation summaries, timestamps, model validation metadata.

The generated blockchain ledger therefore serves as a secure and traceable repository for intrusion auditing and forensic investigation.

#### 3.4.5 Integrity verification

To verify blockchain integrity, the proposed framework recomputes the cryptographic hash of each block and compares it against the stored hash value. Let $H_{i}^{stored\;}$and $H_{i}^{recomputed}$ denote the stored and recomputed hashes, respectively. Integrity verification is performed as:


$$\begin{array}{c} V_{i}=\{\begin{array}{cc} 1, & H_{i}^{stored}=H_{i}^{recomputed}\\ 0, & otherwise \end{array} \end{array}$$ (18)

where *V*_*i*_ = 1 indicates successful integrity validation, *V*_*i*_ = 0 indicates blockchain tampering or data inconsistency.

This validation mechanism guarantees that intrusion logs remain immutable and verifiable throughout the monitoring lifecycle. Thus, the suggested blockchain-inspired tamper-evident validation system offers reliable forensic traceability, secure explainability auditing, and tamper-proof retention of intrusion detection outputs. TAE-IDS improves cybersecurity trust management while guaranteeing the transparency, integrity, and dependability of IDS judgments in distributed network contexts by fusing explainable ensemble intrusion detection with blockchain validation.

#### 3.4.6 Storage overhead

Each blockchain block stores only prediction metadata, SHAP summaries, timestamps, previous hash values, and SHA-256 hash values instead of complete network traffic records. The average block size ranges from approximately 300–600 bytes, depending on the amount of SHAP explanation metadata. Consequently, the storage overhead grows linearly with the number of intrusion predictions while remaining significantly lower than storing raw network packets, making the proposed validation layer suitable for long-term forensic auditing.

#### 3.4.7 Blockchain computational overhead

To evaluate the runtime overhead introduced by the blockchain-inspired tamper-evident validation layer, the execution time of each validation operation was measured. The measured average execution times are summarized in Table 3.

**Table 3:** Computational overhead of the blockchain-assisted validation layer.

Operation	Time (ms)
SHA256 hashing	0.21
Block creation	0.47
Chain verification	0.13
Total blockchain logging latency	0.81

Based on the measured average blockchain logging latency of 0.81 ms per intrusion record, the proposed validation layer achieves an approximate throughput of 1,235 blockchain logging operations per second. These results indicate that the blockchain-assisted validation mechanism introduces only modest computational overhead and is suitable for high-frequency intrusion detection environments without becoming a significant processing bottleneck.

#### 3.4.8 Threat model

The proposed blockchain-inspired tamper-evident validation layer assumes that an attacker may attempt to modify intrusion records after the prediction stage. The representative post-inference attack scenarios considered in this study include:

- Prediction label modification.
- SHAP explanation modification.
- Timestamp alteration.
- Block deletion.
- Block reordering.
- Forged previous-hash insertion.

These representative attack scenarios are used to evaluate the capability of the proposed validation mechanism to detect unauthorized modifications through cryptographic hash verification and previous-hash chain validation.

## 4 Experimental setup

### 4.1 Dataset

Two benchmark intrusion detection datasets, UNSW-NB15 (Moustafa and Slay, 2015) and CICIDS2017 (Sharafaldin et al., 2018), were used in tests to assess the efficacy of the suggested TAE-IDS system. These datasets can be evaluated under both binary and multiclass intrusion detection scenarios due to their different attack categories and heterogeneous traffic patterns.

Because of its varied attack categories and realistic modern network traffic characteristics, UNSW-NB15 was chosen as the main evaluation dataset. 42 traffic features that represent statistical flow characteristics, connection-level statistics, and protocol behavior are included in the dataset. Along with typical traffic samples, it contains nine attack classes: Analysis, Backdoor, DoS, Exploits, Fuzzers, Generic, Reconnaissance, Shellcode, and Worms.

All attack types were combined into a single intrusion class for binary categorization. The initial attack categories were maintained for multiclass classification.

The class-wise distribution of the UNSW-NB15 dataset used in this investigation is shown in Table 4.

**Table 4:** Class-wise distribution of the UNSW-NB15.

Class	Training	Evaluation	Total
Normal	56,000	37,000	93,000
Generic	40,000	18,871	58,871
Exploits	33,393	11,132	44,525
Fuzzers	18,184	6,062	24,246
DoS	12,264	4,089	16,353
Reconnaissance	10,491	3,496	13,987
Analysis	2,000	677	2,677
Backdoor	1,746	583	2,329
Shellcode	1,133	378	1,511
Worms	130	44	174
Total	175,341	82,332	257,673

The predefined training-testing partition of the dataset was preserved to maintain evaluation consistency and reduce the possibility of information leakage.

The CICIDS2017 dataset was used in additional trials to assess TAE-IDS's resilience and generalization capacity (Sharafaldin et al., 2018). In addition to contemporary attack scenarios including DDoS, DoS, Botnet, PortScan, Web attacks, Infiltration, FTP-Patator, SSH-Patator, and Heartbleed attacks, the dataset includes realistic enterprise network traffic.

CICIDS2017 comprises about 2.8 million network flow records and 80 flow-based traffic features that were retrieved using CICFlowMeter. Heartbleed and SQL Injection attacks, in particular, have very few samples, making them extremely unbalanced attack categories. Although CICIDS2017 remains one of the most widely used benchmark datasets for intrusion detection research, previous studies have reported several data quality issues, including labeling inconsistencies, duplicated flow records, and feature-generation errors associated with the original CICFlowMeter processing. Corrected and cleaned variants of the dataset have subsequently been proposed in the literature to address these issues. Nevertheless, the original CICIDS2017 dataset was adopted in this study to ensure fair comparison with the majority of existing intrusion detection literature that continues to use the same benchmark.

Both datasets underwent stratified data partitioning and preprocessing techniques to guarantee accurate evaluation. Additionally, in order to avoid evaluation bias and data leakage, imbalance handling techniques were solely used to the training data.

The proposed TAE-IDS architecture can be thoroughly evaluated across heterogeneous traffic environments, different attack behaviors, and binary and multiclass intrusion detection scenarios by utilizing both UNSW-NB15 and CICIDS2017.

### 4.2 Evaluation metrics

Several statistical and probabilistic measures were used to test the effectiveness of the suggested TAE-IDS architecture in both binary and multiclass intrusion detection scenarios. Because intrusion detection datasets frequently display varied attack distributions and class imbalance, evaluation based only on accuracy may lead to incorrect interpretation. In order to assess classification efficacy, prediction accuracy, and discrimination capacity, additional metrics were used. The primary confusion-matrix-based evaluation metrics are defined as follows (Equations 19–24).


$$\begin{array}{l} Accuracy=\frac{TP+TN}{TP+TN+FP+FN} \end{array}$$ (19)

where *TP*, *TN*, *FP*, and *FN* denote true positives, true negatives, false positives, and false negatives, respectively.

Precision evaluates the reliability of positive intrusion predictions and is computed as:


$$\begin{array}{l} Precision=\frac{TP}{TP+FP} \end{array}$$ (20)

Higher precision values indicate reduced false alarm generation during intrusion detection.

Recall, also referred to as detection rate, measures the ability of the framework to correctly identify intrusion samples:


$$\begin{array}{l} Recall=\frac{TP}{TP+FN} \end{array}$$ (21)

Higher recall values indicate improved attack detection capability and lower missed intrusion rates.

The *F*1-score provides a harmonic balance between precision and recall:


$$\begin{array}{l} F1-Score=\frac{2\times Precision\times Recall}{Precision+Recall} \end{array}$$ (22)

The *F*1-score is particularly useful for evaluating performance under imbalanced intrusion distributions.

The Matthews Correlation Coefficient (MCC) provides a balanced evaluation metric by considering all elements of the confusion matrix:


$$\begin{array}{l} MCC=\frac{TP\times TN-FP\times FN}{\sqrt{\left(TP+FP\right)\left(TP+FN\right)\left(TN+FP\right)\left(TN+FN\right)}} \end{array}$$ (23)

MCC values range from −1 to 1, where 1 indicates perfect classification, 0 represents random prediction, −1 indicates complete disagreement between predictions and actual labels.

Due to its robustness under imbalanced classification settings, MCC provides reliable evaluation for intrusion detection involving minority attack categories.

Log Loss evaluates probabilistic prediction confidence by penalizing incorrect and overconfident classifications:


$$\begin{array}{l} LogLoss=-\frac{1}{N}\sum_{i=1}^{N}\left[y_{i}\log\left(p_{i}\right)+\left(1-y_{i}\right)\log\left(1-p_{i}\right)\right] \end{array}$$ (24)

where *N* denotes the total number of samples, *y*_*i*_ represents the actual label, and *p*_*i*_ denotes the predicted probability.

Lower Log Loss values indicate better probabilistic calibration and prediction reliability.

Threshold-independent discrimination capability was assessed using Receiver Operating Characteristic (ROC) curves and Area Under the Curve (AUC) analysis in addition to confusion-matrix-based measurements. Evaluation metrics for multiclass classification were calculated utilizing a one-vs.-rest approach in conjunction with weighted-average and macro-averaged performance analysis.

In order to keep UNSW-NB15 consistent with recognized benchmarking methodologies, the predetermined training and testing partitions were maintained. Stratified data partitioning was used for CICIDS2017 in order to maintain class distributions. Additionally, only the training data was subjected to SMOTE-based oversampling in order to avoid evaluation bias and data leaking.

### 4.3 Reproducibility and implementation details

See Table 5 for reproducibility and experimental configuration.

**Table 5:** Reproducibility and experimental configuration.

Component	Configuration
Programming language	Python 3.10
Operating system	Ubuntu 22.04 LTS
CPU	Intel Core i7 3.4 GHz
GPU	NVIDIA GTX 1660
RAM	16 GB
Deep learning framework	PyTorch 2.0
Machine learning library	Scikit-learn 1.3
XGBoost version	1.7
Primary random seed	42
Optimizer	Adam
Learning rate	0.001
Batch size	256
Epochs	30
Hidden dimension	96
Dropout	0.4
Activation	Softmax
Loss function	CrossEntropyLoss
Logistic Regression	L2 penalty, max_iter = 2000
Extra Trees	n_estimators = 150, max_depth = 12
XGBoost	n_estimators = 150, max_depth = 3, learning_rate = 0.05
PCA	Fitted using training data only
Chi-square	Fitted using training data only
Min–Max normalization	Fitted using training data only
SMOTE	Applied only to training data
Training/validation/test procedure	**UNSW-NB15:** the predefined training and testing partitions were preserved. **CICIDS2017:** stratified data partitioning was adopted, with the training data further divided into training and validation subsets, while the held-out test set was used exclusively for final performance evaluation

Unless otherwise specified, all experiments reported in Sections 5.1–5.4 were conducted using the primary random seed (42). Section 5.5 presents the statistical robustness analysis based on 10 independent experimental runs using random seeds 0–9.

## 5 Results and evaluation

### 5.1 Detection performance

The UNSW-NB15 and CICIDS2017 benchmark datasets were used to assess the suggested TAE-IDS architecture in binary and multiclass intrusion detection situations. The experimental findings show that the combination of attention-based BiLSTM aggregation, heterogeneous ensemble learning, and uncertainty-aware meta-feature extraction greatly enhances intrusion detection performance in a variety of network contexts.

**Figure 2:**
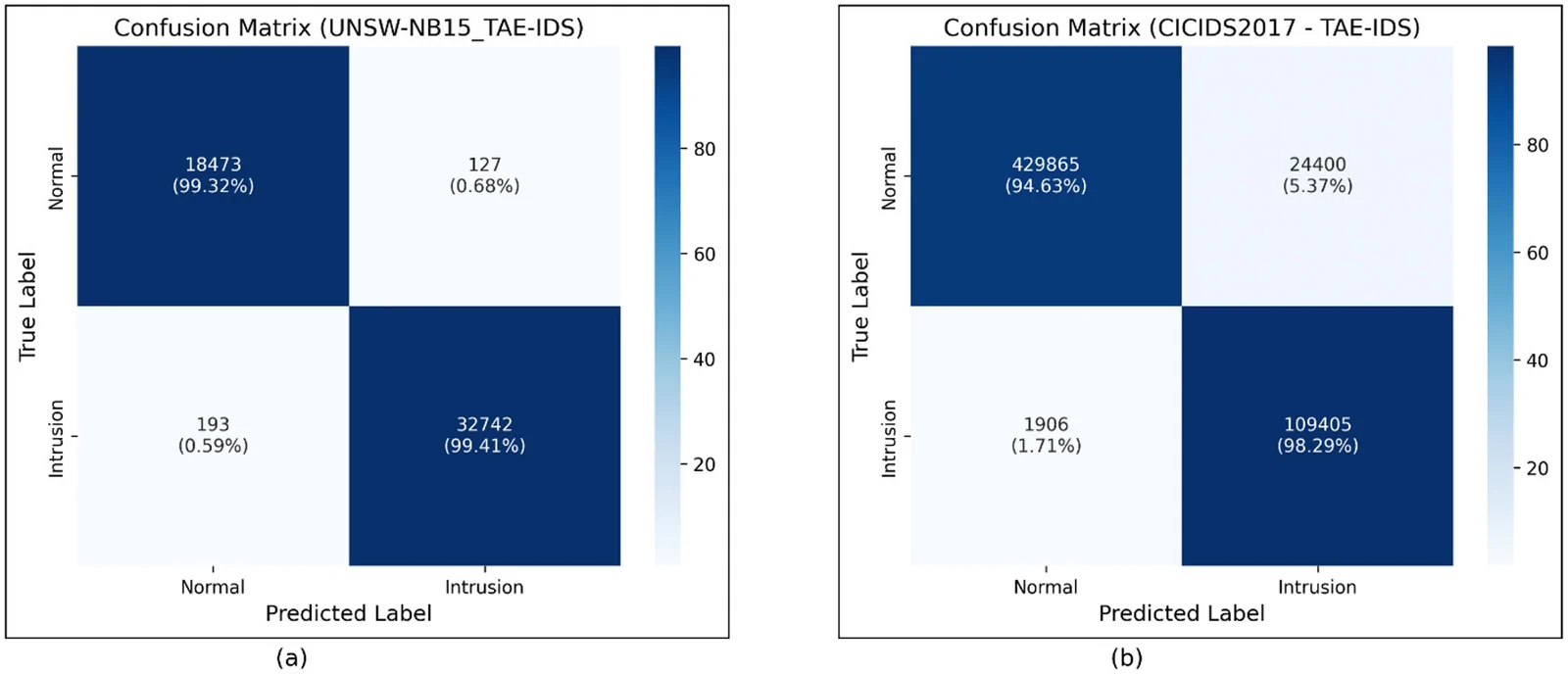
TAE-IDS binary classification confusion matrices of **(a)** UNSW-NB15 and **(b)** CICIDS2017.

The binary classification confusion matrices produced by the suggested TAE-IDS framework on UNSW-NB15 and CICIDS2017 are shown in Figure 2.

The suggested methodology properly identified 99.32% of regular traffic and 99.41% of incursion samples on the UNSW-NB15 dataset. In a similar vein, TAE-IDS correctly classified 98.29% of incursion traffic and 94.63% of benign traffic for CICIDS2017. The dataset's significantly unbalanced attack categories and varied traffic patterns are responsible for the relatively lower benign categorization rate seen in CICIDS2017.

The efficiency of the uncertainty-aware attention-based ensemble technique in learning discriminative intrusion patterns while retaining good generalization capabilities across various traffic environments is demonstrated by the high true positive rates and decreased false alarm generation.

**Figure 3:**
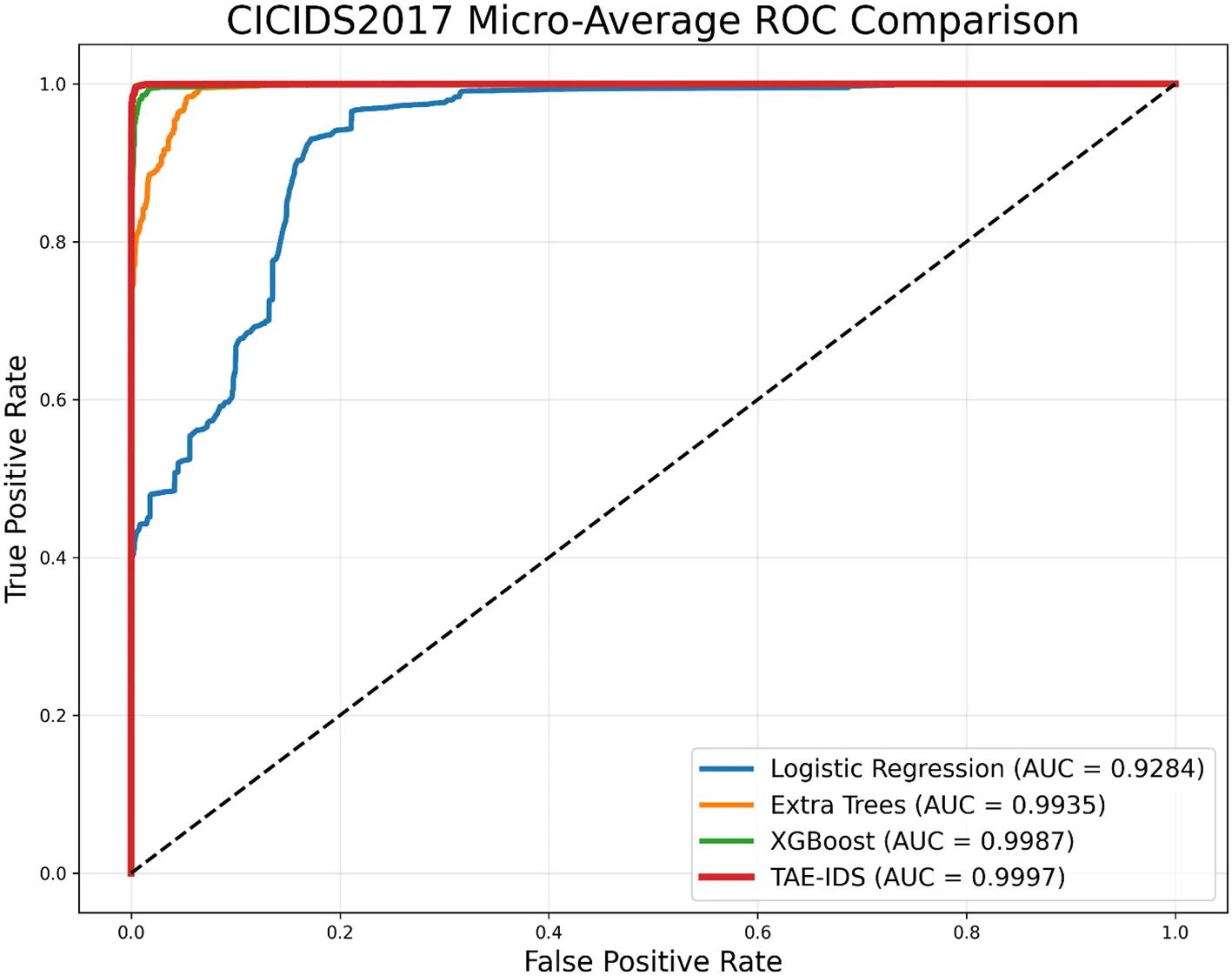
Multiclass ROC comparison of LR, ET, XGB, and TAE-IDS on CICIDS2017.

The ROC-based performance comparison of XGB, Extra Trees, LR, and the suggested TAE-IDS framework on the CICIDS2017 multiclass intrusion detection challenge is shown in Figure 3.

With a micro-average AUC value of 0.9997, the suggested framework outperformed the baseline classifiers and demonstrated the highest discrimination capabilities. Because of its linear decision boundaries, LR showed very poor discrimination ability, ET and XGB showed excellent non-linear learning performance. However, by using adaptive attention-guided meta-learning, the suggested TAE-IDS framework considerably enhanced classification separability. The results of binary classification performance comparison on UNSW-NB15 and CICIDS2017 is shown in Table 6.

**Table 6:** Binary classification performance comparison on UNSW-NB15 and CICIDS2017.

Model	UNSW Acc (%)	CICIDS Acc (%)	MCC (UNSW)	MCC (CICIDS)	Log Loss (UNSW)	Log Loss (CICIDS)
LR	92.87	84.01	0.8507	0.6409	0.1685	0.2862
Extra Trees	97.97	86.2	0.956	0.6814	0.1621	0.3643
XGB	87.16	88.82	0.7317	0.6155	0.2834	0.2308
TAE-IDS	99.37	95.35	0.9863	0.8693	0.02	0.1091

**Figure 4:**
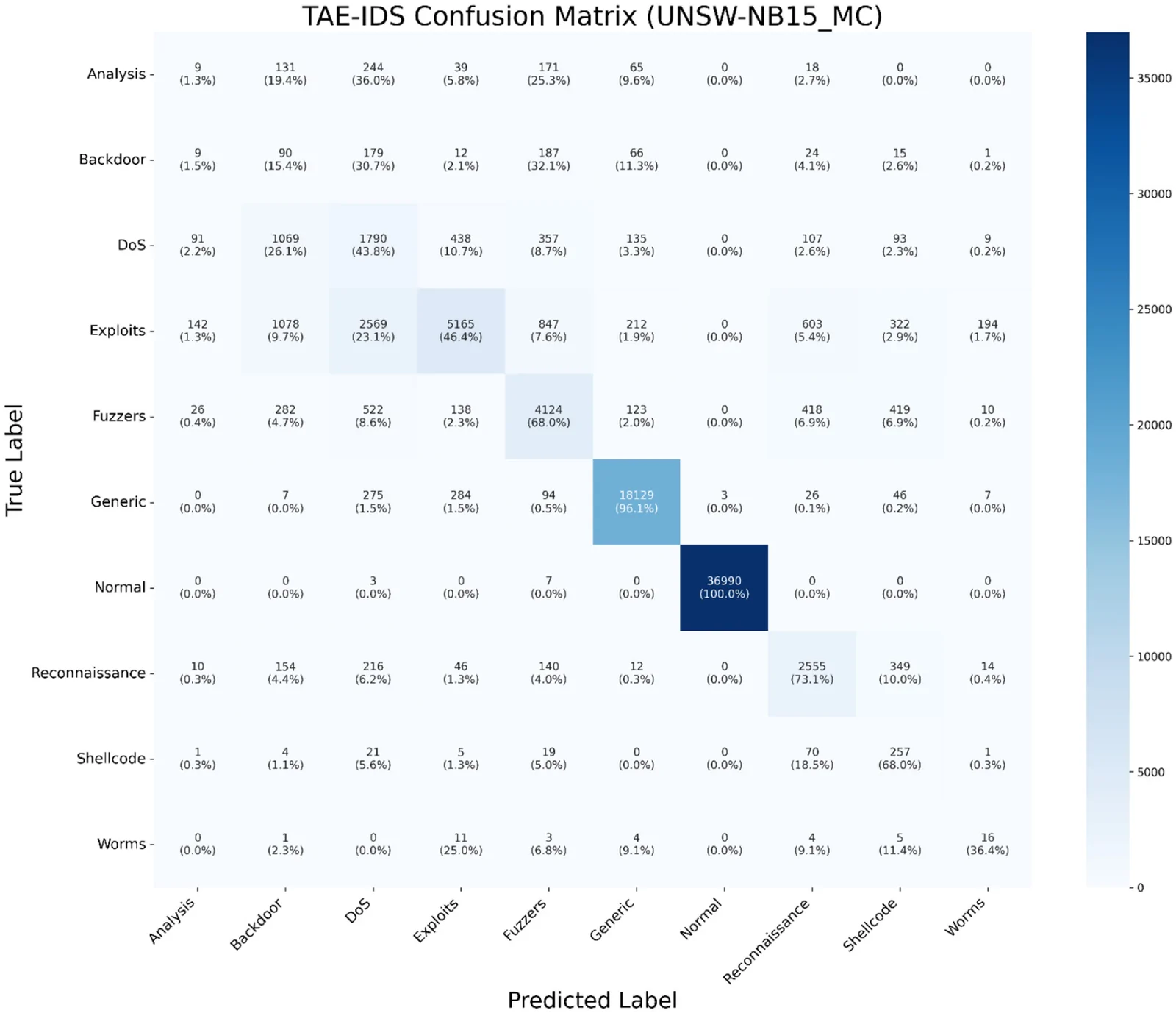
Multiclass confusion matrix of TAE-IDS on UNSW-NB15.

**Figure 5:**
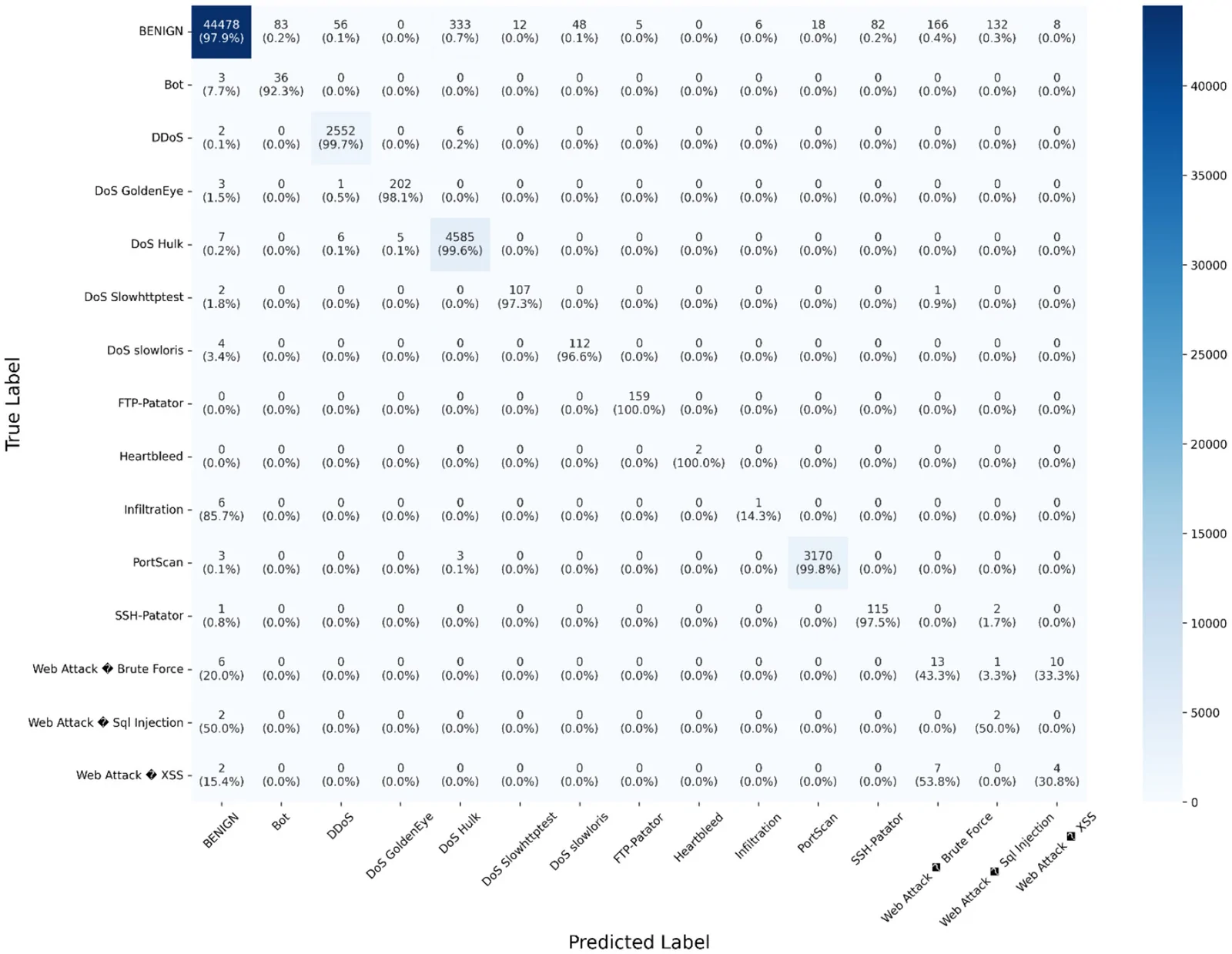
Multiclass confusion matrix of TAE-IDS on CICIDS2017.

Figures 4, 5 present the multiclass confusion matrices of the proposed TAE-IDS framework for the UNSW-NB15 and CICIDS2017 datasets, respectively. The confusion matrices demonstrate high classification accuracy for the majority of attack categories with relatively limited inter-class misclassification. Most prediction errors occur among minority attack classes having very few training instances, indicating that class imbalance remains the primary challenge rather than limitations of the proposed architecture.

Across both datasets, the suggested TAE-IDS architecture continuously demonstrated better Accuracy, Precision, Recall, *F*1-score, MCC, and Log Loss performance. The enhancement can be ascribed to the complementing combination of adaptive BiLSTM attention fusion, uncertainty-aware meta-feature modeling, and heterogeneous base learners.

The multiclass classification performance of the proposed TAE-IDS framework on the UNSW-NB15 and CICIDS2017 datasets is presented in Table 7. In addition, Tables 8–10 provide a comprehensive per-class and overall multiclass performance analysis, demonstrating the capability of the proposed framework to accurately differentiate fine-grained attack categories under highly imbalanced traffic conditions.

**Table 7:** Multiclass classification performance comparison of TAE-IDS on UNSW-NB15 and CICIDS2017.

Model	UNSW accuracy (%)	CICIDS accuracy (%)	MCC (UNSW)	MCC (CICIDS)
LR	63.62	51.33	0.5682	0.3945
Extra Trees	69.66	82.49	0.6383	0.6783
XGB	74.05	95.14	0.6836	0.8525
TAE-IDS	76.5	98.18	0.703	0.9497

**Table 8:** Per-class performance of TAE-IDS on UNSW-NB15 (multiclass).

Class	Precision	Recall (detection rate)	*F*1-Score	False alarm rate	PR-AUC	Support
Analysis	0.0717	0.1533	0.0977	0.0208	0.137	535
Backdoor	0.0788	0.103	0.0893	0.011	0.0732	466
DoS	0.3205	0.6175	0.422	0.0887	0.3104	3,271
Exploits	0.7463	0.5097	0.6057	–	–	8,905
Fuzzers	0.5663	0.5717	0.569	–	–	4,849
Generic	0.991	0.9774	0.9841	–	–	11,774
Normal	0.914	0.8714	0.8922	–	–	18,600
Reconnaissance	0.7456	0.7112	0.728	–	–	2,798
Shellcode	0.3243	0.4768	0.3861	0.0059	0.3195	302
Worms	0.2045	0.2571	0.2278	0.0007	0.1236	35

False alarm rate (FAR) and PR-AUC are reported for the most challenging minority attack classes to highlight the detection behavior under severe class imbalance. The overall Macro PR-AUC across all attack categories is summarized in this table.

**Table 9:** Per-class performance of TAE-IDS on CICIDS2017 (multiclass).

Class	Precision	Recall (detection rate)	*F*1-score	False alarm rate	PR-AUC	Support
BENIGN	0.999	0.986	0.9924	0.004	0.9992	45,427
Bot	0.4444	0.9231	0.6	0.0008	0.6126	39
DDoS	0.9853	0.9969	0.9911	0.0007	0.9993	2,560
DoS GoldenEye	0.9621	0.9854	0.9736	0.0001	0.9865	206
DoS Hulk	0.9439	0.9946	0.9686	0.0052	0.9971	4,603
DoS Slowhttptest	0.9068	0.9727	0.9386	0.0002	0.9072	110
DoS slowloris	0.8702	0.9828	0.9231	0.0003	0.9735	116
FTP-Patator	0.9693	0.9937	0.9814	0.0001	0.9992	159
Heartbleed	1	1	1	0	1	2
Infiltration	0.5	0.1429	0.2222	0	0.0741	7
PortScan	0.9947	0.9981	0.9964	0.0003	0.9981	3,176
SSH-Patator	0.9435	0.9915	0.9669	0.0001	0.9836	118
Web attack–Brute Force	0.0946	0.4667	0.1573	0.0024	0.2546	30
Web attack–SQL Injection	0.0303	0.75	0.0583	0.0017	0.5081	4
Web attack–XSS	0.1379	0.3077	0.1905	0.0004	0.1289	13

**Table 10:** Overall multiclass performance metrics of TAE-IDS.

Metric	UNSW-NB15	CICIDS2017
Macro precision	0.4963	0.7188
Macro recall	0.5249	0.8328
Macro *F*1-score	0.5002	0.7307
Weighted precision	0.8028	0.9917
Weighted recall	0.7630	0.9873
Weighted *F*1-score	0.7756	0.9892
Balanced accuracy	0.5249	0.8328
Macro PR-AUC	0.5057	0.7615

Per-Class Multiclass Performance Analysis. Tables 8–10 present the detailed per-class performance evaluation of the proposed TAE-IDS framework. In addition to precision, recall, and *F*1-score, false alarm rate (FAR), detection rate, PR-AUC, support, macro-averaged metrics, weighted-averaged metrics, and balanced accuracy are reported to provide a comprehensive assessment of multiclass intrusion detection performance.

For the UNSW-NB15 dataset, the proposed TAE-IDS achieved high detection performance for majority attack categories, including Generic, Normal, and Reconnaissance, while comparatively lower performance was observed for minority classes such as Backdoor, Analysis, Shellcode, and Worms. These classes contain substantially fewer training instances (e.g., Worms: 35 samples and Shellcode: 302 samples), leading to severe class imbalance, which limits the model's ability to learn sufficiently discriminative feature representations. Nevertheless, their false alarm rates remained low, indicating that misclassifications primarily resulted from limited sample availability rather than excessive false positive predictions.

On the CICIDS2017 dataset, TAE-IDS demonstrated consistently high precision, recall, and PR-AUC for major attack categories including DDoS, PortScan, FTP-Patator, DoS Hulk, DoS GoldenEye, and BENIGN traffic. Relatively lower performance was observed for extremely rare attack categories such as Infiltration, Web Attack–SQL Injection, Web Attack–Brute Force, and Web Attack–XSS, which contain only a few training samples. Despite these highly imbalanced classes, the proposed attention-based ensemble maintained high weighted *F*1-score (0.9892) and balanced accuracy (0.8328), demonstrating robust overall multiclass generalization.

Overall, the experimental results show that TAE-IDS maintains good generalization performance across varied cybersecurity datasets while offering robust intrusion detection capacity under both binary and multiclass scenarios.

#### 5.1.1 Comparison with recent state-of-the-art methods

To further evaluate the effectiveness of the proposed TAE-IDS framework and to provide a quantitative comparison with representative published intrusion detection methods, the proposed framework was compared with recent state-of-the-art IDS models that reported results on the UNSW-NB15 or CICIDS2017 benchmark datasets whenever comparable experimental results were available. The comparison includes literature-reported results, reproduced results, and results obtained in this work to clearly distinguish the origin of each reported performance value.

As shown in Table 11, the proposed TAE-IDS achieves competitive intrusion detection performance compared with representative recent IDS methods that reported results on the UNSW-NB15 or CICIDS2017 benchmark datasets. In addition to maintaining strong classification performance, the proposed framework uniquely integrates heterogeneous attention-based meta-ensemble learning, SHAP-based explainability, and blockchain-assisted tamper-evident validation within a unified architecture. Since the reported results originate from different studies with varying preprocessing strategies, train-test partitions, feature engineering methods, and evaluation protocols, the numerical comparison should be interpreted as a comparative reference rather than a strictly controlled experimental benchmark. Nevertheless, the comparison indicates that the proposed TAE-IDS achieves competitive intrusion detection performance while additionally providing SHAP-based explainability, forensic traceability, and blockchain-assisted trust validation within a unified framework.

**Table 11:** Comparison of the proposed TAE-IDS with recent state-of-the-art intrusion detection methods.

Method	Technique	UNSW-NB15	CICIDS2017	Result source
Thakkar and Lohiya (2023)	Bagging-based deep neural network	*F*1-score: 98.78%	*F*1-score: 99.86%	Literature (reported)
Hashmi et al. (2024)	CNN + BiLSTM + attention + RF	Accuracy: 99.56%	–	Literature (reported)
Umer et al. (2025)	Multi-Head Attention Transformer	Accuracy: 93.00%	–	Literature (reported)
Pawar et al. (2025)	Ensemble ML + blockchain	–	Accuracy: 99.00%	Literature (Reported)
HAMC-ID (Antony Joseph Raj and Madiajagan, 2025)	Attention-based meta-ensemble	Accuracy: 98.94%	Accuracy: 95.27%	Reproduced
TAE-IDS (proposed)	Attention-based meta-ensemble + SHAP + blockchain	Accuracy: 99.37%	Accuracy: 95.35%	This work

Performance values for the compared methods were obtained directly from their respective publications. Only studies reporting quantitative results on the UNSW-NB15 and/or CICIDS2017 benchmark datasets were included in this comparison. Since preprocessing procedures, feature engineering strategies, train-test partitions, feature selection methods, and evaluation protocols differ across studies, these results are presented for comparative reference rather than as strictly controlled experimental benchmarks. Results labeled reproduced and this work were obtained using the experimental methodology described in this paper.

### 5.2 Explainability analysis

To improve transparency and interpretability in intrusion detection decisions, the proposed TAE-IDS framework incorporates a SHAP-based explainability module that provides both global and local explanations for intrusion predictions. Unlike conventional black-box intrusion detection systems that only generate classification outputs, the proposed framework enables security analysts to understand how uncertainty-aware ensemble meta-features influence the final intrusion detection decision.

**Figure 6:**
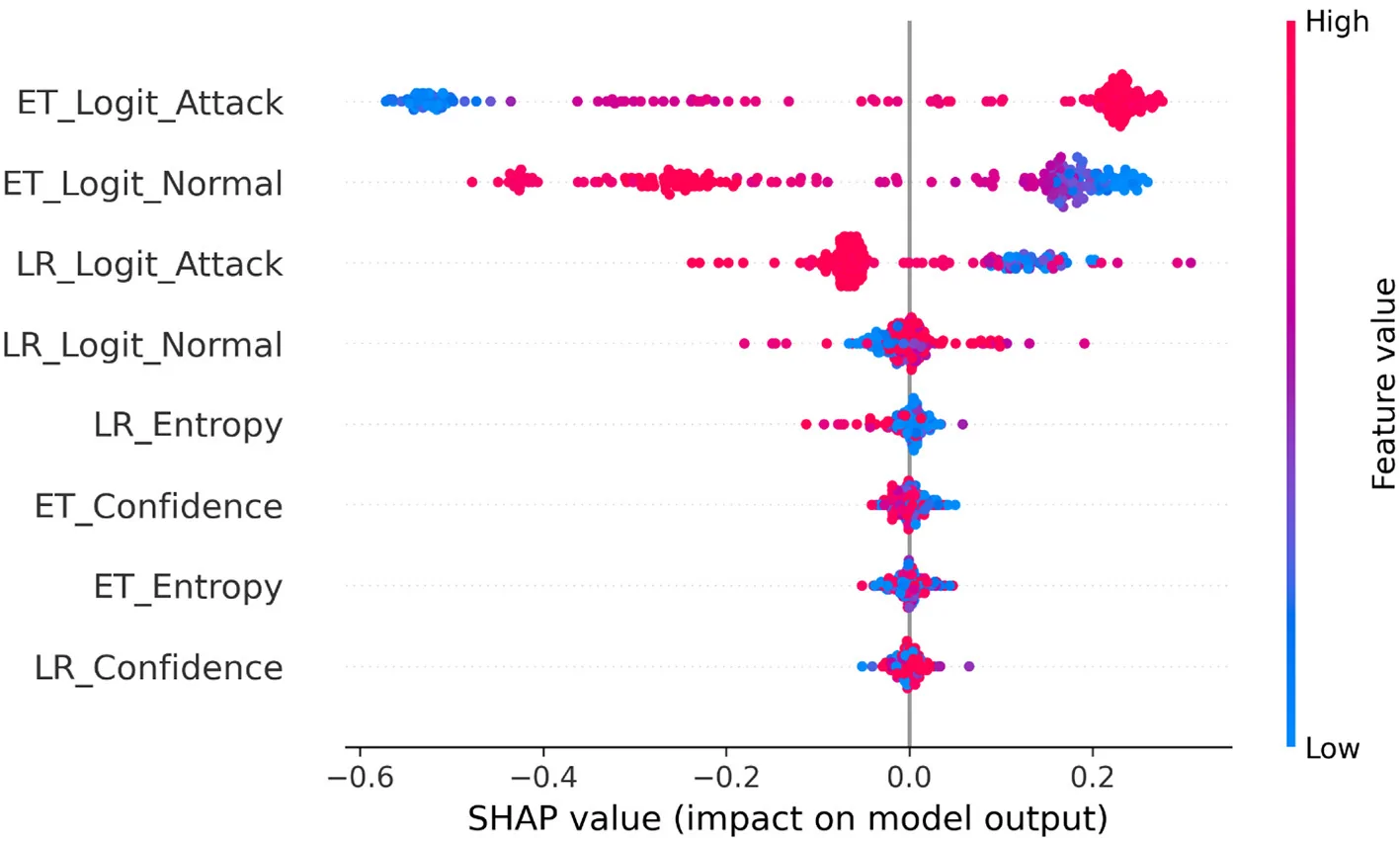
SHAP summary plot for meta-feature importance.

Figure 6 presents the SHAP summary plot of the uncertainty-aware meta-features used by the proposed TAE-IDS framework on the UNSW-NB15 dataset. The feature labels correspond to the logits, confidence scores, and entropy values generated by the heterogeneous base learners. The results indicate that ET_Logit_Attack and ET_Logit_Normal exhibit the highest SHAP importance values, demonstrating that the Extra Trees classifier contributes most significantly to the final ensemble decision. The remaining meta-features, including Logistic Regression logits, confidence scores, and entropy values, provide complementary information that enhances the robustness of the attention-based meta-classifier.

Positive SHAP values increase the contribution of a meta-feature toward the predicted intrusion class, whereas negative SHAP values reduce its influence. The distribution of SHAP values demonstrates that the uncertainty-aware ensemble effectively captures discriminative intrusion patterns while maintaining transparent and interpretable decision behavior.

**Figure 7:**
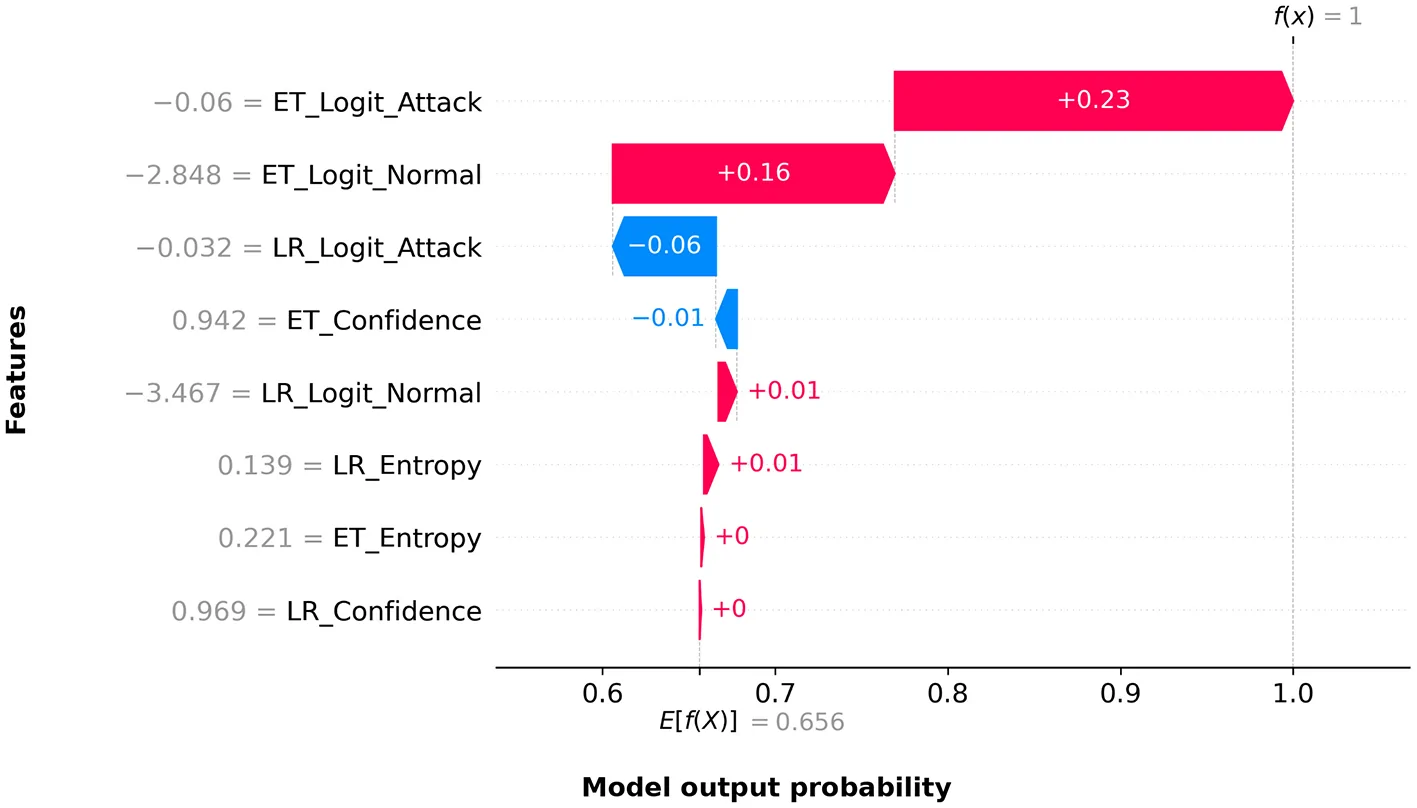
SHAP waterfall plot for local intrusion prediction explanation.

The SHAP waterfall graphic for a single intrusion prediction produced by the suggested TAE-IDS architecture is shown in Figure 7.

Figure 7 presents the SHAP waterfall plot for an individual intrusion prediction, providing an instance-level explanation of the decision-making process. Positive SHAP values increase the likelihood of the predicted attack class, whereas negative SHAP values reduce it. In this example, ET_Logit_Attack and ET_Logit_Normal contribute most strongly to the final prediction, indicating that the Extra Trees base learner plays the dominant role for this particular network flow. In contrast, the Logistic Regression meta-features make comparatively smaller contributions, illustrating how the attention-based meta-classifier integrates complementary evidence from multiple base learners.

Similar explainability behavior was observed for the multiclass intrusion detection experiments on both the UNSW-NB15 and CICIDS2017 datasets. Across different attack categories, the SHAP analysis consistently identified the ensemble logit representations as the most influential meta-features, followed by classifier confidence scores and entropy-based uncertainty measures. These findings indicate that the proposed attention-based meta-classifier primarily relies on informative logit representations while utilizing confidence and uncertainty information to improve the robustness of the final intrusion decision. The interpretability contribution of the main meta-features taken from the heterogeneous ensemble framework is summarized in Table 12.

**Table 12:** SHAP-based meta-feature interpretation in TAE-IDS.

Rank	Feature	Mean|SHAP|importance
1	ET_Logit_Normal	0.154487481
2	ET_Logit_Attack	0.145435737
3	LR_Logit_Attack	0.021821232
4	LR_Logit_Normal	0.02099449
5	ET_Confidence	0.020314828
6	LR_Entropy	0.012533344
7	LR_Confidence	0.01156007
8	ET_Entropy	0.009758776

The SHAP-based explainability analysis demonstrates that TAE-IDS not only achieves strong intrusion detection performance but also provides transparent reasoning regarding ensemble behavior, uncertainty estimation, and adaptive decision formation. By identifying the contributions of the heterogeneous base learners and their associated uncertainty-aware meta-features, the proposed framework enhances analyst trust, supports forensic investigation, and improves the interpretability of intrusion detection decisions in both binary and multiclass cybersecurity environments.

#### 5.2.1 Feature-level explainability using TreeSHAP

While the SHAP analysis presented in Figures 6, 7 provides explainability at the ensemble level by quantifying the contributions of the uncertainty-aware meta-features, an additional feature-level SHAP analysis was performed to identify the original network traffic attributes responsible for the intrusion decision. Since the Extra Trees classifier exhibited the highest SHAP importance among the heterogeneous base learners (Figures 6, 7), it was selected for feature-level explainability using TreeSHAP. This complementary analysis enables direct attribution of intrusion predictions to the underlying traffic characteristics while providing a more comprehensive interpretation of the proposed TAE-IDS framework.

**Figure 8:**
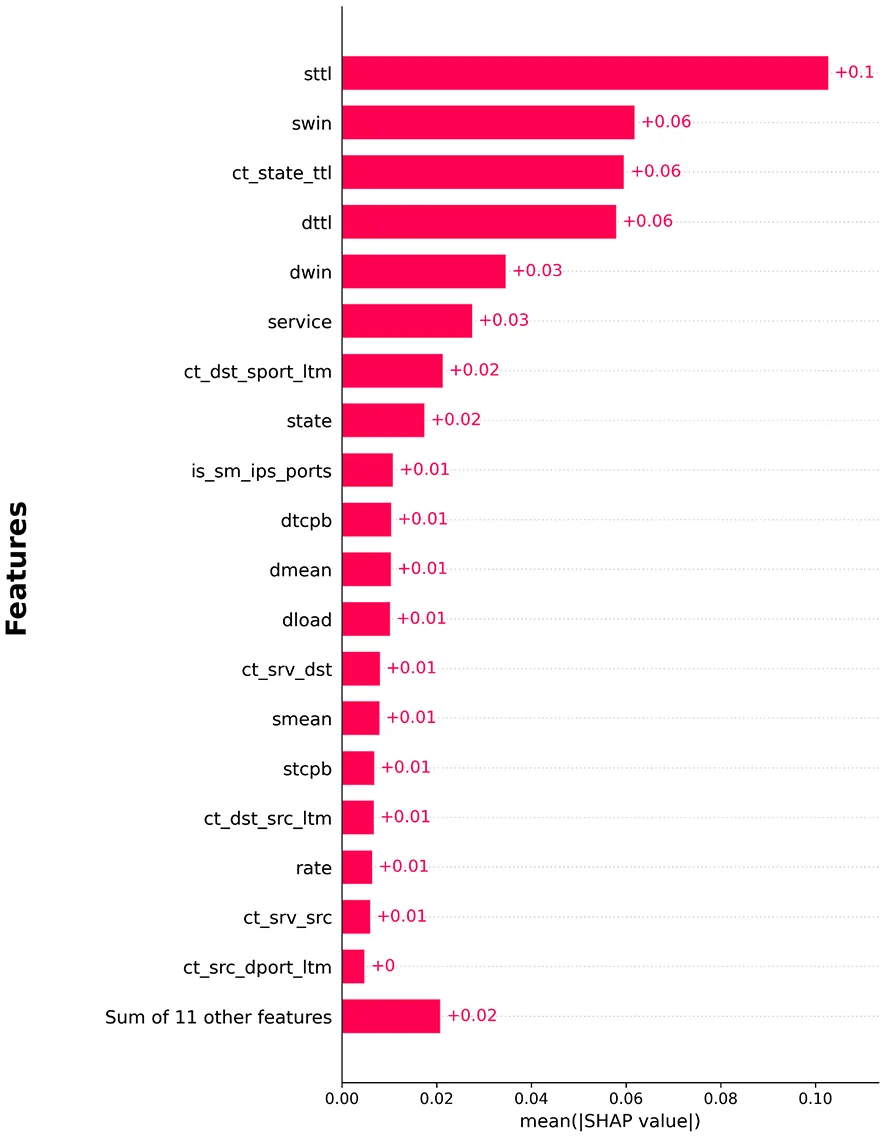
Global SHAP feature importance of original network traffic features using the Extra Trees base learner.

Figure 8 presents the global SHAP importance scores computed from the original network traffic feature space. The results indicate that sttl, swin, ct_state_ttl, dttl, dwin, and service are the most influential features contributing to intrusion detection. These findings are consistent with the ensemble-level SHAP analysis, where the Extra Trees classifier was identified as the dominant contributor to the final prediction. The identified features represent important characteristics of network communication, including packet time-to-live values, TCP window sizes, connection state information, and service behavior, thereby providing direct traffic-level attribution for the intrusion decision.

**Figure 9:**
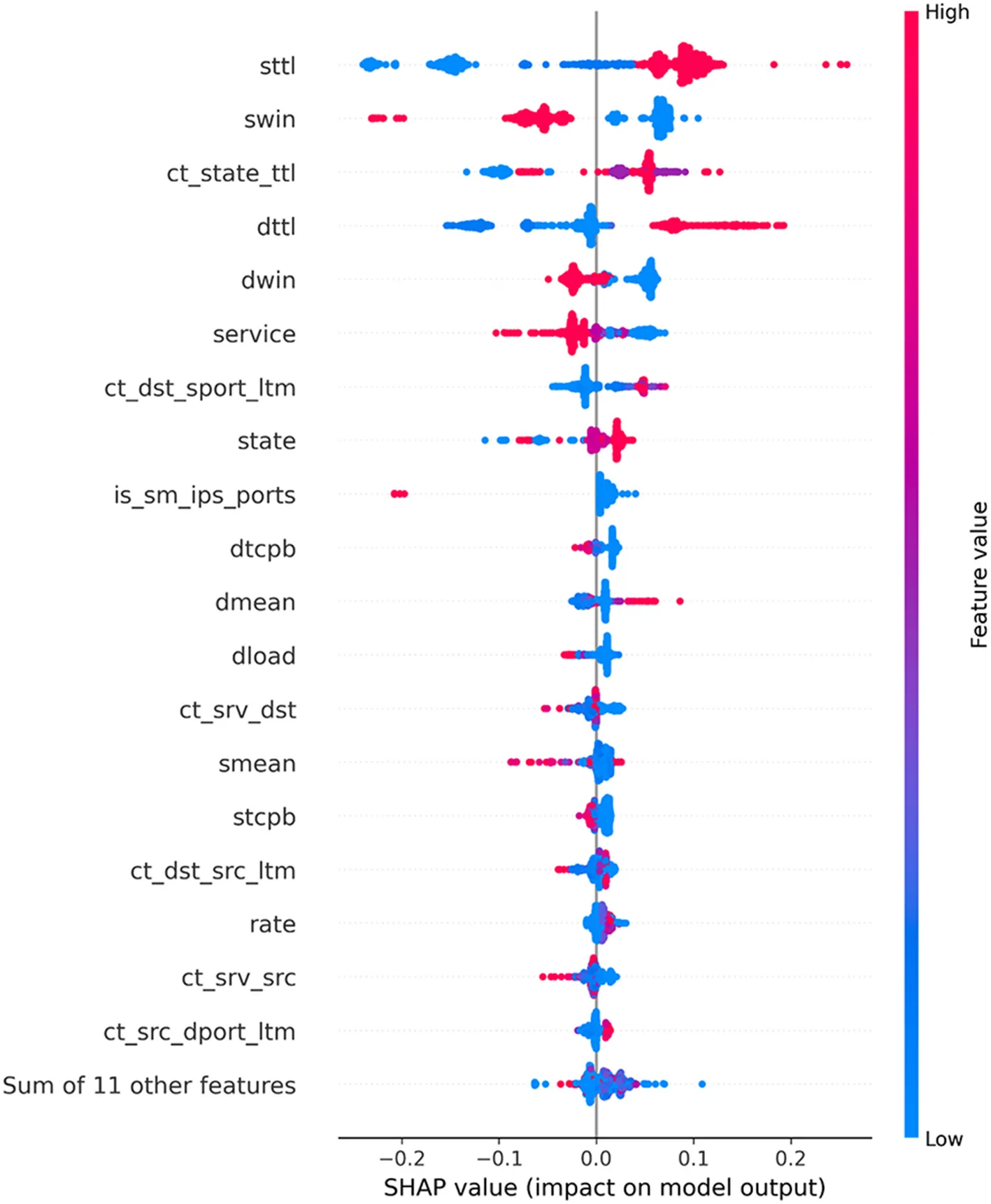
SHAP beeswarm plot showing the distribution of original network traffic feature contributions.

Figure 9 illustrates both the magnitude and direction of feature contributions across multiple network flows. Features with higher SHAP values increase the likelihood of intrusion detection, whereas negative SHAP values decrease the prediction score. The color distribution further demonstrates how different feature values influence the classifier, providing comprehensive insight into feature behavior over the entire dataset.

**Figure 10:**
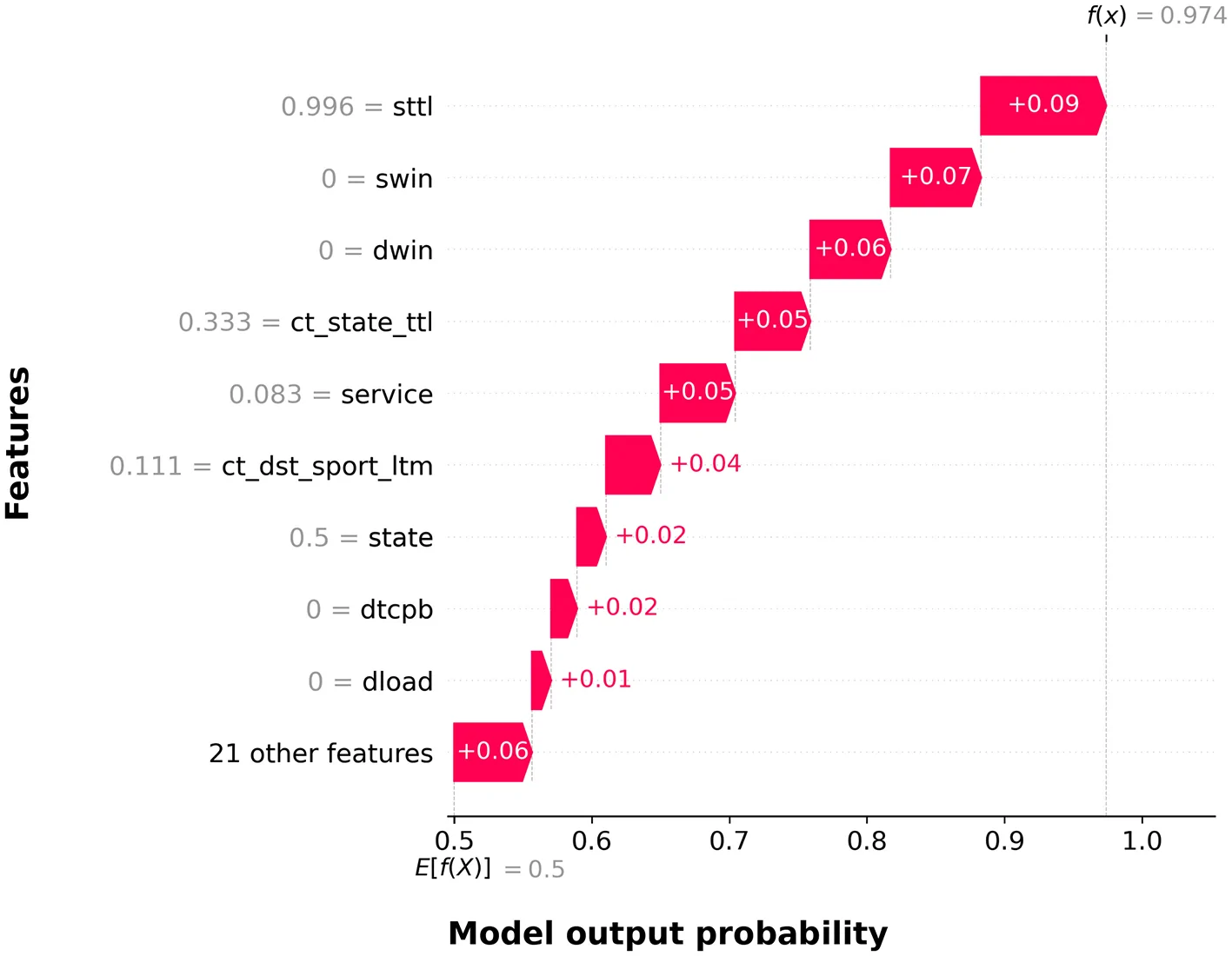
SHAP waterfall plot for an individual intrusion prediction based on original network traffic features.

Figure 10 provides a local explanation for an individual intrusion prediction. The waterfall plot demonstrates how the original network traffic attributes collectively contribute to the final prediction. In this example, sttl, swin, dwin, ct_state_ttl, and service provide the largest positive contributions toward the predicted intrusion class, illustrating the network characteristics responsible for the classifier's decision.

The proposed TAE-IDS framework therefore provides complementary explainability at two levels. First, SHAP analysis of the uncertainty-aware meta-features explains how the attention-based ensemble integrates the predictions, confidence scores, and uncertainty information generated by the heterogeneous base learners to produce the final intrusion decision. Second, TreeSHAP analysis of the most influential Extra Trees base learner identifies the original network traffic features that drive individual intrusion predictions. Together, these complementary analyses provide both ensemble-level transparency and traffic-level attribution, offering a comprehensive explanation of intrusion decisions that enhances interpretability, analyst trust, forensic investigation, and practical cybersecurity deployment.

### 5.3 Blockchain tampering validation

To evaluate the robustness of the proposed blockchain-assisted validation layer, six representative tampering attacks were experimentally implemented after intrusion prediction. These attacks included prediction label modification, SHAP metadata alteration, timestamp manipulation, block deletion, block reordering, and forged previous-hash insertion. Following each attack, blockchain integrity verification recomputed the SHA-256 hash of every block and validated the previous-hash chain. As shown in Table 13, all six tampering scenarios resulted in integrity verification failure, indicating that every unauthorized modification was successfully detected. These results demonstrate that the proposed blockchain validation mechanism provides effective tamper-evident protection for intrusion records and associated explainability metadata, thereby enhancing forensic integrity and trustworthiness.

**Table 13:** Blockchain tampering validation results.

Attack scenario	Modified component	Integrity verification	Tampering detected
Prediction label modification	Prediction label	Failed	Yes
SHAP metadata modification	SHAP explanation	Failed	Yes
Timestamp modification	Timestamp	Failed	Yes
Block deletion	Blockchain structure	Failed	Yes
Block reordering	Previous hash link	Failed	Yes
Forged previous hash	Hash chain	Failed	Yes

### 5.4 Blockchain validation

The proposed TAE-IDS system incorporates a blockchain-inspired tamper-evident validation mechanism for tamper-proof storing of intrusion predictions and explainability metadata to improve the dependability and forensic credibility of intrusion detection outputs. The resulting prediction outputs, confidence levels, timestamps, and explanation summaries are safely recorded into successive blockchain blocks using SHA-256 cryptographic hashing following intrusion classification and SHAP-based interpretation.

**Figure 11:**
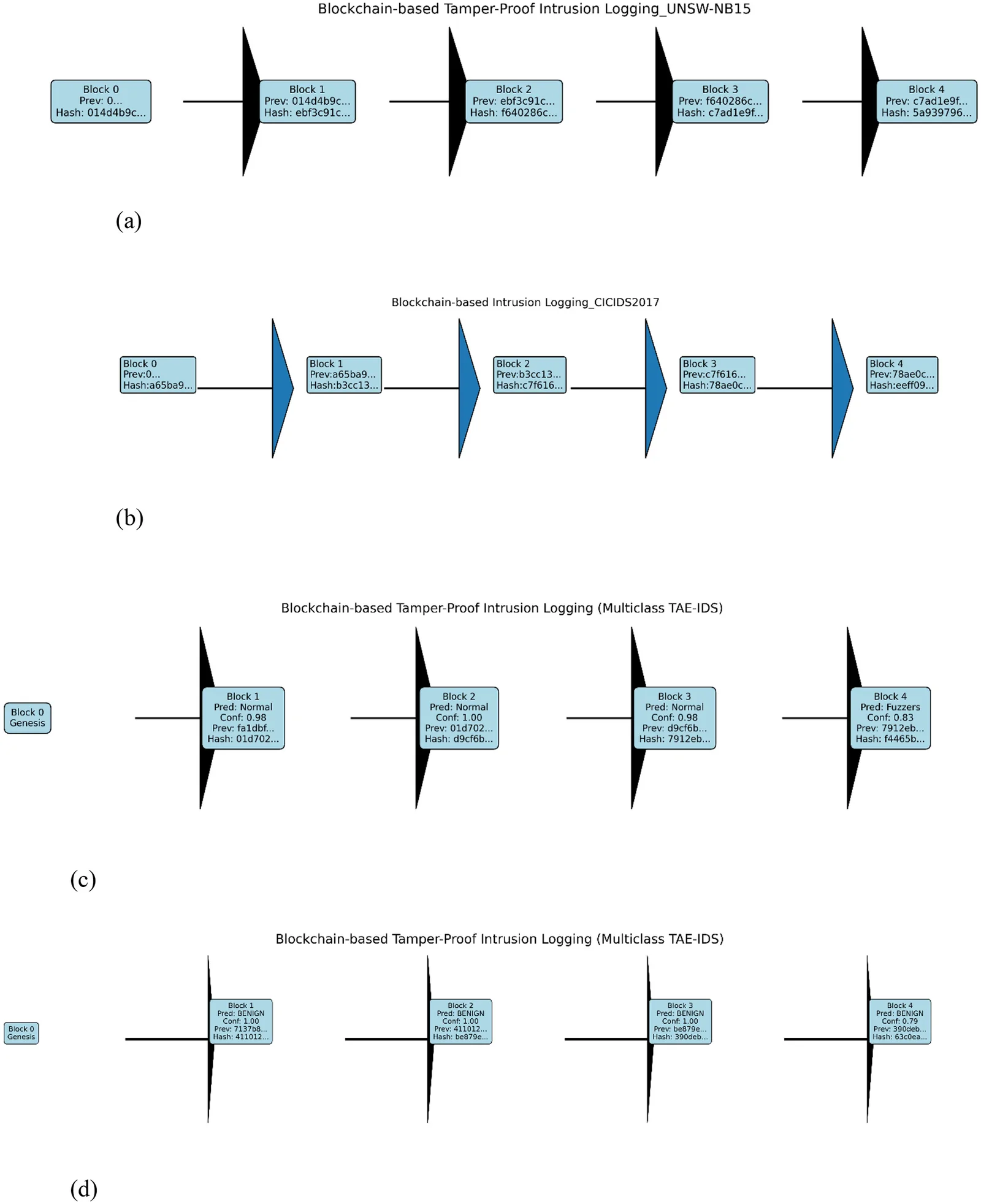
**(a)** Blockchain-secured intrusion logging for binary classification on UNSW-NB15. **(b)** Blockchain-secured intrusion logging for binary classification on CICIDS2017. **(c)** Blockchain-secured multiclass intrusion logging on UNSW-NB15. **(d)** Blockchain-secured multiclass intrusion logging on CICIDS2017.

The blockchain-secured logging procedure for binary intrusion detection on the UNSW-NB15 and CICIDS2017 datasets is shown in Figures 11a, b, respectively. Sequential integrity is maintained across the blockchain ledger by storing prediction information in each generated block together with the previous hash reference and the freshly generated current hash.

Successful cryptographic linking between successive intrusion recordings is demonstrated by the blockchain chain. The generated blockchain records, for instance, have chained hash associations like:

- Previous Hash: fa1dbfb944... → Current Hash: 01d702940d...
- Previous Hash: 01d702940d... → Current Hash: d9cf6beb72...
- Previous Hash: d9cf6beb72... → Current Hash: 7912eb1c90...

Through SHA-256 hashing, these sequential dependencies verify that every blockchain block is mathematically connected to its predecessor. As a result, tamper detection and secure forensic auditing are made possible by the instantaneous invalidation of all subsequent blockchain relationships caused by any illegal modification to recorded incursion records.

The blockchain validation results for multiclass intrusion detection on UNSW-NB15 and CICIDS2017 are shown in Figures 11c, d, respectively. The multiclass blockchain records not only maintain safe block chaining but also the attack-category predictions and corresponding confidence levels produced by the TAE-IDS framework.

The multiclass blockchain results show that diverse intrusion categories including Normal, BENIGN, and Fuzzers, along with corresponding confidence scores and SHAP explanation summaries, may be successfully stored securely. While more uncertain traffic conditions produced relatively lower confidence levels, such as 0.8293 and 0.5122, several intrusion events produced confidence values near 1.0 for highly separable traffic patterns. These uncertainty-aware forecasts are safely maintained by the blockchain layer, which prevents post-generation alteration.

The blockchain validation statistics gathered during the experimental evaluation are compiled in Table 14.

**Table 14:** Blockchain validation results of TAE-IDS on UNSW-NB15 and CICIDS2017.

Dataset	Total blocks	Integrity status	Hash algorithm
UNSW-NB15	51,536	Verified	SHA-256
CICIDS2017	565,577	Verified	SHA-256

All created blockchain records successfully passed cryptographic validation without chain inconsistency or tampering detection, according to the integrity verification findings. The suggested blockchain-secured intrusion recording framework's scalability and resilience in high-volume cybersecurity monitoring environments are further demonstrated by the substantial number of validated blockchain blocks.

All things considered, the proposed TAE-IDS framework's credibility, transparency, and forensic reliability are greatly improved by the blockchain-inspired tamper-evident validation module. The proposed approach combines explainable intrusion detection with unchangeable cryptographic audit trails to provide reliable cybersecurity decision traceability and secure evidence preservation in both binary and multiclass intrusion detection scenarios.

### 5.5 Statistical robustness analysis

The performance results presented in Tables 6–10 correspond to the primary experimental run, whereas this subsection evaluates the statistical robustness of the proposed framework over 10 independent experimental runs using different random seeds.

To evaluate the robustness and reproducibility of the proposed TAE-IDS framework, the complete experimental pipeline was repeated using 10 independent random seeds (0–9) while maintaining the same training, validation, and testing configuration. For each experimental run, model initialization, optimization, and training were independently repeated. The mean and standard deviation of Accuracy, Macro *F*1-score, and Matthews Correlation Coefficient (MCC) were computed for Logistic Regression (LR), Extra Trees (ET), XGBoost (XGB), and the proposed TAE-IDS. Furthermore, paired *t*-tests, Wilcoxon signed-rank tests, and bootstrap confidence intervals were employed to determine whether the performance improvements achieved by TAE-IDS over the baseline classifiers were statistically significant. The statistical robustness results are summarized in Table 15.

**Table 15:** Statistical robustness of TAE-IDS and baseline models over 10 independent random seeds (mean ± standard deviation).

Model	Accuracy (%)	Macro *F*1-score	MCC
Logistic Regression	92.84 ± 0.08	0.9239 ± 0.0008	0.8501 ± 0.0015
Extra Trees	97.26 ± 0.23	0.9702 ± 0.0025	0.9404 ± 0.0049
XGBoost	87.10 ± 0.17	0.8459 ± 0.0020	0.7298 ± 0.0044
TAE-IDS	99.12 ± 0.19	0.9905 ± 0.0020	0.9810 ± 0.0040

As shown in Table 15, the proposed TAE-IDS consistently achieved the highest Accuracy, Macro *F*1-score, and MCC across all 10 experimental runs while exhibiting very low standard deviations. The small performance variations observed across different random seeds indicate that the proposed attention-based meta-ensemble learning framework is stable, reproducible, and insensitive to random initialization. These results demonstrate that the superior performance of TAE-IDS is consistently maintained rather than being dependent on a particular experimental run.

In Table 16 the paired *t*-test and Wilcoxon signed-rank test confirmed that the improvements achieved by TAE-IDS over Logistic Regression, Extra Trees, and XGBoost were statistically significant (*p* < 0.05) across all comparisons. Furthermore, the bootstrap 95% confidence intervals for the mean performance improvements excluded zero in every case, indicating that the observed improvements are statistically reliable and unlikely to have occurred due to random variation. Collectively, these statistical analyses provide strong evidence that the proposed TAE-IDS framework consistently and significantly outperforms Logistic Regression, Extra Trees, and XGBoost while maintaining stable and reproducible performance across repeated experimental runs.

**Table 16:** Statistical significance analysis of TAE-IDS compared with baseline classifiers over 10 independent random seeds.

Baseline	Mean accuracy improvement (%)	Paired *t*-test (*p*-value)	Wilcoxon (*p*-value)	Bootstrap 95% *CI*	Significant
Logistic Regression	6.27	< 0.001	0.001953	[6.12%, 6.40%]	Yes (*p* < 0.05)
Extra Trees	1.86	< 0.001	0.001953	[1.73%, 1.98%]	Yes (*p* < 0.05)
XGBoost	12.02	< 0.001	0.001953	[11.84%, 12.20%]	Yes (*p* < 0.05)

### 5.6 Computational complexity analysis

Because the proposed TAE-IDS architecture integrates attention-based meta-learning, SHAP explainability analysis, and blockchain-inspired tamper-evident validation, it has more computational overhead than traditional machine learning-based intrusion detection systems. Nonetheless, the ensuing rise in computing complexity is still controllable and offers notable enhancements in forensic dependability, transparency, and trust-preserving intrusion auditing.

Different computational properties are displayed by the heterogeneous base classifiers. LR uses computational complexity to carry out linear optimization:


$$\begin{array}{c} O\left(nd\right) \end{array}$$

where *n* denotes the number of training samples, *d* denotes the feature dimensionality.

The ET classifier performs randomized ensemble tree construction with complexity:


$$\begin{array}{c} O\left(Kndlogn\right) \end{array}$$

where *K* denotes the number of ensemble trees.

Similarly, the XGB classifier performs iterative gradient-boosted tree optimization with computational complexity:


$$\begin{array}{c} O\left(Tnd\right) \end{array}$$

where *T* denotes the number of boosting iterations.

The adaptive BiLSTM-based attention aggregation module introduces additional sequential learning overhead for contextual meta-feature reasoning. The approximate complexity of the BiLSTM attention module is:


$$\begin{array}{c} O\left(nh^{2}\right) \end{array}$$

where *h* denotes the hidden-state dimensionality of the BiLSTM network.

The SHAP explainability module introduces additional computational overhead because feature attribution requires repeated model evaluations. The computational complexity depends on the selected SHAP approximation method and the number of evaluated features. Although SHAP increases the *post-hoc* explanation time compared with conventional black-box intrusion detection systems, the additional overhead is acceptable for forensic analysis and security auditing. However, SHAP computation is still possible for forensic cybersecurity auditing and offline intrusion analysis.

The blockchain-inspired tamper-evident validation layer carries out linearly complex integrity verification, chained block creation, and SHA-256 hashing:


$$\begin{array}{c} O\left(b\right) \end{array}$$

where *b* denotes the total number of blockchain intrusion records.

The significant improvements in explainability, transparency, integrity preservation, and tamper-proof forensic validation outweigh the higher computational overhead of the suggested TAE-IDS framework compared to conventional ensemble IDS architectures. The suggested framework offers reliable cybersecurity decision support through integrated explainable AI and blockchain-assisted validation processes, in contrast to traditional black-box intrusion detection systems that only concentrate on classification performance.

The framework's lightweight blockchain hashing operations and ensemble base learners' minimal complexity make it useful for contemporary cybersecurity contexts even with the incorporation of several security-focused modules. As a result, the suggested TAE-IDS system strikes a fair compromise between computational viability, interpretability, secure validation, and intrusion detection capacity.

#### 5.6.1 Computational performance and real-time feasibility

To further evaluate the practical deployment feasibility of the proposed TAE-IDS framework, the execution time of each major processing stage was experimentally measured. Unlike the theoretical computational complexity discussed in the previous subsection, this section reports the observed runtime of the model training, inference, SHAP explainability, and blockchain validation components. The measured computational performance is summarized in Table 17.

**Table 17:** Computational performance and real-time feasibility analysis of the proposed TAE-IDS.

Component	Average time
Training time (base classifiers: LR, ET, XGBoost)	30.00 s
Training time (TAE-IDS meta-classifier)	709.45 s
Inference time (TAE-IDS)	1.3162 s
SHAP explanation time (200 evaluation samples)	20.00 s
Average SHAP explanation time	100 ms/sample
SHA-256 hashing	0.21 ms
Block creation	0.47 ms
Chain verification	0.13 ms
Total blockchain logging	0.81 ms
Storage overhead	300–600 bytes/block
Blockchain logging throughput	1,235 validation operations/s

The experimental timing analysis demonstrates that the proposed TAE-IDS framework is suitable for practical intrusion detection applications. The heterogeneous base classifiers required approximately 30.00 s for model training, whereas the attention-based BiLSTM meta-classifier required 709.45 s because of iterative deep learning optimization. Since model training is performed offline, the higher training cost does not affect operational deployment. During testing, the proposed framework completed inference in 1.3162 s, indicating efficient prediction performance for online intrusion detection. The SHAP explainability module required approximately 20.00 s to generate explanations for 200 evaluation samples, corresponding to an average explanation time of 100 ms per sample. Although SHAP introduces additional *post-hoc* computational overhead, it provides valuable model interpretability and forensic transparency without affecting the real-time prediction pipeline. The blockchain-assisted validation mechanism introduced only 0.81 ms of additional latency per intrusion record, including 0.21 ms for SHA-256 hashing, 0.47 ms for block creation, and 0.13 ms for chain verification. Furthermore, the lightweight validation layer required only 300–600 bytes per blockchain block and achieved an approximate throughput of 1,235 validation operations per second, demonstrating that secure blockchain-assisted logging can be integrated with minimal computational overhead. Overall, these results indicate that the proposed TAE-IDS framework achieves an effective balance between detection accuracy, explainability, blockchain-assisted trust validation, and computational efficiency, making it suitable for real-time intrusion detection applications.

## 6 Ablation study

An ablation study was conducted to evaluate the contribution of the major components incorporated into the proposed TAE-IDS framework. Specifically, the study investigates the influence of ensemble meta-feature extraction, lightweight attention pooling, BiLSTM-based attention aggregation, and blockchain-assisted tamper-evident validation on intrusion detection performance.

Controlled experiments were performed on the UNSW-NB15 and CICIDS2017 benchmark datasets. Individual components were incrementally added to the overall TAE-IDS architecture to assess their impact on Accuracy, Precision, Recall, *F*1-score, Matthews Correlation Coefficient (MCC), Log Loss, end-to-end execution time, and inference latency. The corresponding results are summarized in Table 18.

**Table 18:** Ablation study of the proposed TAE-IDS framework.

Variant	Accuracy (%)	Precision	Recall	*F*1-score	MCC	Log Loss	End-to-end execution time (s)	Inference time (s)
Extra Trees	97.9703	0.9746	0.9941	0.9843	0.956	0.1621	23.8192	2.7016
Ensemble meta features	99.2956	0.9947	0.9943	0.9945	0.9847	0.0241	9.4203	0.0069
Attention pooling (lightweight)	98.69	0.9919	0.9876	0.9898	0.9718	0.0364	290.68	0.8963
BiLSTM without attention	99.348	0.9965	0.9933	0.9949	0.9859	0.0196	187.3413	1.3372
Full TAE-IDS w/o blockchain	99.3868	0.996	0.9944	0.9952	0.9867	0.0185	200.9951	1.4614
Complete TAE-IDS	99.3868	0.996	0.9944	0.9952	0.9867	0.0185	203.479	1.4614

The reported end-to-end execution time includes model training, inference, SHAP explanation generation, and blockchain-assisted validation where applicable. Because blockchain validation is executed only after intrusion prediction, the additional execution time observed for the complete TAE-IDS framework reflects post-inference logging and integrity verification rather than additional model training.

The ablation results demonstrate the progressive contribution of each architectural component. The standalone Extra Trees classifier achieved the lowest overall performance, indicating that a single classifier is insufficient for accurately modeling complex intrusion patterns. Incorporating ensemble meta-feature representations substantially improved Accuracy, MCC, and Log Loss while maintaining low inference latency, demonstrating the effectiveness of combining complementary classifier outputs.

To address the effectiveness of the proposed fusion strategy, a lightweight Attention Pooling baseline was implemented using the same ensemble meta-features. Although this simpler model achieved a competitive Accuracy of 98.69%, its performance remained below that of the proposed BiLSTM-based fusion model, which achieved 99.35% Accuracy without attention removal and 99.39% Accuracy in the complete TAE-IDS framework. Furthermore, the proposed model achieved a higher MCC (0.9867 vs. 0.9718) and lower Log Loss (0.0185 vs. 0.0364), indicating improved discrimination capability and better-calibrated prediction probabilities. The lightweight Attention Pooling model required considerably lower computational time (290.68 s training and 0.8963 s inference), whereas the BiLSTM-attention module incurred additional computational cost in exchange for consistently superior detection performance.

The BiLSTM-based aggregation module further enhanced contextual learning by modeling dependencies among ensemble meta-features. Removing the attention mechanism slightly reduced Accuracy and MCC, confirming that adaptive attention weighting contributes to more effective feature integration and improved intrusion discrimination.

The blockchain-assisted tamper-evident validation layer introduced only a modest computational overhead while preserving identical classification performance. The end-to-end execution time increased slightly from 200.9951 s to 203.4790 s after blockchain integration, whereas inference latency remained virtually unchanged. This demonstrates that the proposed blockchain validation mechanism improves forensic traceability, tamper resistance, and trustworthiness without significantly affecting intrusion detection efficiency.

Overall, the complete TAE-IDS framework achieved the best balance between detection performance, explainability, secure validation, and computational reliability. The ablation analysis confirms that each architectural component contributes complementary capabilities, while the comparison with the lightweight Attention Pooling baseline further demonstrates that the proposed BiLSTM-attention fusion provides measurable improvements in detection accuracy and predictive reliability despite its additional computational cost.

## 7 Discussion

The results of the experiment demonstrate that the proposed TAE-IDS framework successfully integrates intrusion detection capability, blockchain-secured trust maintenance, and explainability-driven decision analysis into a unified cybersecurity architecture. Compared to conventional intrusion detection systems, which are primarily focused on classification accuracy, the proposed method emphasizes transparent reasoning and safe forensic validation of intrusion predictions.

The SHAP-based explainability module significantly improves transparency by enabling interpretable reasoning for both local and global incursion decisions. Because traditional deep learning and ensemble-based intrusion detection systems often function as black-box models, it may be difficult for cybersecurity analysts to understand why particular traffic events are classified as harmful. Conversely, the proposed explainability layer makes use of SHAP summary plots and waterfall visualizations to facilitate feature-level contribution analysis. The results of the experiment showed that ensemble logit representations, confidence ratings, and entropy-based uncertainty features have a substantial impact on intrusion prediction behavior. This transparency increases analyst trust and facilitates forensic investigation by enabling accurate interpretation of uncertainty-aware intrusion decisions in both binary and multiclass systems.

The blockchain-inspired tamper-evident validation layer enhances cybersecurity trust management by providing immutable logging and tamper-proof intrusion record storage. Conventional IDS logging systems are still vulnerable to unauthorized security data tampering, erasure, or manipulation after intrusion detection. The proposed blockchain-assisted validation technique, which employs SHA-256 cryptographic hashing and chained block verification, addresses these limitations. Effective integrity retention over extended intrusion recording efforts was confirmed by experimental results on the UNSW-NB15 and CICIDS2017 datasets. The generated blockchain chains, which remained fully verifiable without inconsistency or hash mismatch, exhibited secure auditability of intrusion detection outputs and dependable forensic traceability.

Combining explainability with blockchain validation makes intelligent intrusion detection systems much more dependable. The explainability layer enables transparent decision-making, while the blockchain layer guarantees the secure storage and verification of intrusion evidence. Together, these components improve the transparency, interpretability, and integrity of intrusion detection under the evaluated benchmark datasets. Further validation using operational network environments is required before assessing deployment in large-scale or high-risk network scenarios. Despite the significant advantages, integrating SHAP analysis with blockchain validation adds additional processing overhead when compared to conventional intrusion detection systems.

SHAP explanation generation improves interpretability computation during inference, but blockchain-secured logging requires additional cryptographic hashing and chain validation processes. Because blockchain validation is performed only after model inference, it does not affect the training of the attention-based meta-classifier. The additional execution time observed in the ablation study is attributable solely to post-inference blockchain logging and integrity verification. As a result, the suggested framework has slightly more processing complexity and storage overhead than traditional ensemble-based IDS models.

Nonetheless, given the significant advancements in explainability, forensic dependability, integrity preservation, and cybersecurity trust management, the observed overhead is still acceptable. Therefore, for contemporary cybersecurity environments where transparency, auditability, and tamper-proof intrusion validation are essential operational needs, the trade-off between computing expense and secure, reliable intrusion detection is warranted.

Overall, the benchmark-based experimental results demonstrate that integrating explainable AI with blockchain-assisted tamper-evident validation enhances transparency, forensic traceability, and intrusion detection performance while preserving adaptive detection capability. Additional evaluation using live operational environments is required to assess long-term robustness and deployment feasibility.

Compared with our previously published HAMC-ID framework, the proposed TAE-IDS not only preserves the adaptive attention-based ensemble architecture but also introduces explainability and secure validation capabilities. The integration of SHAP enables transparent reasoning for intrusion decisions, while the tamper-evident validation layer enhances the integrity and traceability of IDS outputs, which may facilitate future deployment in practical cybersecurity environments.

Although the proposed attention-based BiLSTM consistently improves intrusion detection performance over direct ensemble aggregation, the meta-feature sequence remains relatively short. Consequently, lightweight fusion mechanisms, such as gated multilayer perceptrons or attention-based fusion networks, may achieve comparable performance with lower computational complexity. The proposed BiLSTM was selected because it enables contextual bidirectional fusion of heterogeneous uncertainty-aware classifier representations before attention-based aggregation while maintaining consistent performance improvements in the ablation study. Nevertheless, a comprehensive comparison with alternative lightweight fusion strategies remains an important direction for future research.

### 7.1 Limitations and future deployment considerations

Although the proposed TAE-IDS framework demonstrates strong performance on the UNSW-NB15 and CICIDS2017 benchmark datasets, several limitations should be acknowledged. First, the experimental evaluation was conducted using publicly available benchmark datasets, which may not fully represent the diversity, scale, and continuously evolving characteristics of operational network environments. Consequently, the reported performance may differ when deployed in real-world enterprise networks.

Second, the benchmark datasets may exhibit dataset bias and traffic aging, as attack behaviors, benign traffic patterns, and network protocols continuously evolve over time. Periodic model retraining and adaptation would therefore be necessary to maintain detection performance against emerging cyber threats.

Third, although the proposed framework incorporates SHAP-based explainability and blockchain-assisted tamper-evident validation, it has not yet been validated using live network traffic or continuous online deployment. Future work will evaluate the framework using real-time packet capture and streaming network environments to further assess deployment feasibility.

In addition, the current framework does not explicitly address adversarial machine learning attacks, where carefully crafted inputs may influence the predictions of machine learning models. Improving robustness against adversarial examples remains an important direction for future research.

Furthermore, the proposed framework assumes relatively stable data distributions during training and evaluation. In practical cybersecurity environments, concept drift caused by evolving attack strategies and changing network behaviors may gradually reduce model performance. Future work will investigate continual learning and adaptive model updating to improve long-term robustness.

The present evaluation also focuses primarily on benchmark flow-based datasets. The effectiveness of the proposed framework on highly encrypted network traffic, where payload information is unavailable, requires further investigation.

Finally, although the blockchain-assisted validation mechanism introduces only modest computational overhead, the scalability of the proposed validation layer under large-scale distributed deployments involving multiple validation nodes and substantially higher traffic volumes has not yet been evaluated. Future work will investigate distributed blockchain architectures and large-scale real-time deployment scenarios to further improve scalability and operational robustness.

Another limitation relates to the CICIDS2017 benchmark dataset itself. Previous studies have identified labeling inconsistencies, duplicated flow records, and feature-generation errors in the original dataset, and corrected or cleaned variants have subsequently been proposed in the literature to address these issues. Although the original benchmark was intentionally retained in this work to facilitate comparison with existing studies, future work will evaluate the proposed TAE-IDS framework using corrected or cleaned variants of CICIDS2017 and additional real-world cybersecurity datasets to further assess robustness and generalization.

Despite these limitations, the benchmark-based experimental results demonstrate that the proposed TAE-IDS framework effectively integrates attention-based meta-ensemble learning, SHAP-based explainability, and blockchain-assisted tamper-evident validation, providing a strong foundation for future real-world validation studies.

## 8 Conclusion

This paper presented TAE-IDS, an attention-based explainable intrusion detection framework that integrates heterogeneous ensemble learning, adaptive BiLSTM-based attention aggregation, blockchain-secured tamper-proof validation, and SHAP-driven explainability analysis into a single cybersecurity architecture. The main shortcomings of current intrusion detection systems, such as black-box decision behavior, opaque logic, and the susceptibility of intrusion logs to unauthorized manipulation, were addressed by the suggested architecture.

The suggested system exhibits strong intrusion detection capabilities in both binary and multiclass classification scenarios, according to experimental evaluation carried out on the UNSW-NB15 and CICIDS2017 benchmark datasets. While uncertainty-aware meta-feature modeling enhances classification robustness under mixed network traffic conditions, the adaptive ensemble learning mechanism successfully captures complicated non-linear incursion patterns.

By offering both local incursion prediction interpretation and global feature-importance analysis, the SHAP-based explainability module greatly improves transparency. The explainability layer increases analyst trust, interpretability, and forensic reasoning capacity by helping cybersecurity analysts comprehend how ensemble logits, confidence scores, and entropy-based uncertainty features influence intrusion decisions.

Additionally, the blockchain-inspired tamper-evident validation module uses chained block verification and SHA-256 cryptographic hashing to ensure integrity preservation, unchangeable intrusion logging, and secure forensic auditing. The produced blockchain structures improved cybersecurity trust management and secure evidence preservation by successfully maintaining tamper-proof incursion recordings across extensive logging processes.

The proposed work's main contribution is the seamless integration of blockchain-assisted trust validation, adaptive attention-based ensemble intrusion detection, and explainable AI into a single IDS framework. The proposed TAE-IDS framework integrates intrusion detection, explainability, transparency, and blockchain-assisted tamper-evident validation within a unified architecture evaluated on benchmark datasets, in contrast to traditional intrusion detection systems that mainly concentrate on detection performance alone.

The experimental results indicate that the additional computational overhead introduced by SHAP explainability and blockchain-assisted validation is modest under the evaluated benchmark settings while providing improved transparency and forensic traceability.

Future research will concentrate on developing real-time streaming intrusion detection for high-speed network environments and applying the suggested blockchain validation layer within actual distributed blockchain infrastructures. For large-scale intelligent network defense systems, additional research may look into scalable trust-aware cybersecurity architectures, federated intrusion detection, and lightweight explainability optimization.

## Data Availability

Publicly available datasets were analyzed in this study. This data can be found here: UNSW-NB15 (https://research.unsw.edu.au/projects/unsw-nb15-dataset); CICIDS2017 (http://cicresearch.ca/CICDataset/CIC-IDS-2017/Dataset/CIC-IDS-2017).
